# L-leucine-incorporated 3D-printed SilMA hydrogel scaffolds promote calvarial defect repair with PLOD2-associated collagen remodeling

**DOI:** 10.1016/j.mtbio.2026.103513

**Published:** 2026-07-30

**Authors:** QiaoYu Zhang, ZiJie An, Lianzong Hang, JingYu Liu, Chen Gu, Jia Zhu, YaWei Zhang, Lei Wang, Wenhui Hu, Tianming Wang, Xin Zhang, Yue Mao, Rui Zhao, Yongqiang Zhang, Kun Zhu

**Affiliations:** a Department of Orthopaedics, The First Affiliated Hospital of Bengbu Medical University, Bengbu, 233000, China; b Department of Geriatrics, The First Affiliated Hospital of Bengbu Medical University, Bengbu, 233000, China; c Department of Basic Teaching of General Practice, The Second Affiliated Hospital of Bengbu Medical University(The Second Clinical College of Bengbu Medical University), Bengbu, 233000, China; d Department of Orthopaedics, Geriatric Hospital of Nanjing Medical University, Nanjing, 210000, China; e Institute of Digital and Intelligent Healthcare, Bengbu Medical University, Bengbu, 233000, China; f College of Biotechnology and Pharmaceutical Engineering, Nanjing Tech University, Nanjing, 210000, China; g Department of Orthopedics, Nanjing First Hospital, Nanjing Medical University, Nanjing, 210000, China; h Nanjing Medical University, Nanjing, 210000, China; i Department of Orthopaedic Surgery, Institute of Digital Medicine, Nanjing First Hospital, Nanjing Medical University, Nanjing, 210000, China

**Keywords:** L-leucine, Silk fibroin, 3D printing, Bone defect, Osteogenesis, Angiogenesis

## Abstract

Effective repair of bone defects requires scaffolds that not only match defect geometry and provide mechanical support, but also support both osteogenic and angiogenic processes during regeneration. Here, a photocurable hydrogel ink integrating methacrylated silk fibroin (SilMA) with L-leucine (L-Leu) is developed for 3D printing of architected scaffolds with programmable pore structures. L-Leu incorporation enhances the compressive performance and surface wettability of the hydrogel without compromising print fidelity, thereby improving the interfacial microenvironment for cell attachment and tissue remodeling. In vitro, SilMA@L-Leu exhibits good cytocompatibility, promotes bone marrow–derived mesenchymal stem cell proliferation and osteogenic differentiation, and enhances the angiogenic activity of human umbilical vein endothelial cells. Among the tested formulations, SilMA@L-Leu containing 300 ng mL^−1^ L-Leu showed the most balanced osteogenic and angiogenic performance and was therefore selected for mechanistic and in vivo evaluation. In a rat critical-size calvarial defect model, this scaffold markedly promotes new bone formation, collagen deposition, and mineralized tissue development. Mechanistically, siRNA-mediated silencing of procollagen-lysine, 2-oxoglutarate 5-dioxygenase 2 (PLOD2) attenuated the scaffold-induced osteogenic effects, suggesting the involvement of PLOD2-associated collagen matrix remodeling in the pro-osteogenic activity of SilMA@L-Leu. These findings identify SilMA@L-Leu as a 3D-printable, small-molecule-incorporated scaffold with promise for bone defect repair.

## Graphical abstract

**Figure:**
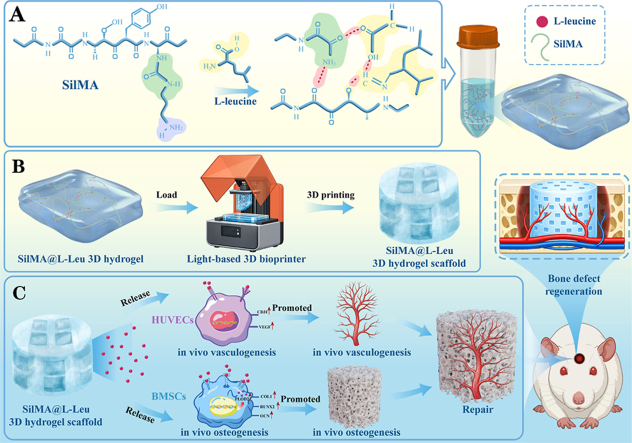

## 1 Introduction

Large-volume or geometrically complex bone defects, particularly those arising in load-bearing or anatomically intricate sites, remain a major challenge in orthopaedic reconstruction [1]. Once bone loss exceeds a critical threshold, endogenous healing is often insufficient to restore structural integrity and function within a clinically relevant timeframe [2]. Although debridement, bone marrow stimulation, and autograft/allograft transplantation are widely used, these approaches are limited by donor scarcity, immunological and infection-related risks, suboptimal morphological and mechanical matching, and uncertain long-term stability [3]. There is therefore a persistent need for material systems that provide early structural support while continuously directing cell behavior and tissue remodeling throughout regeneration [4].

Silk fibroin (SF) has attracted broad interest in biomedical applications because of its favorable biocompatibility, processability, tunable degradation, and intrinsic mechanical merits, making it a promising scaffold material for bone tissue engineering [5]. Silk-based hydrogels can partially recapitulate the hydrated three-dimensional extracellular matrix environment and thereby support cell adhesion, migration, and tissue infiltration [6]. However, conventional silk hydrogels typically rely on physical gelation or standard chemical crosslinking, which limits control over network architecture and mechanical performance. As a result, it remains challenging to simultaneously achieve shape fidelity, adjustable mechanics, and degradation behavior aligned with tissue regeneration, particularly in complex defects or mechanically demanding settings [7]. Methacrylated silk fibroin (SilMA) addresses these limitations by introducing photo-crosslinkable moieties that enable rapid network formation under mild conditions, thereby expanding the design space for crosslinking density, printing precision, and mechanical tunability [8].

Beyond structural support, an ideal scaffold should provide sustained bioinstructional cues that promote bone marrow–derived mesenchymal stem cell (BMSC) attachment, proliferation, and osteogenic differentiation while concurrently supporting endothelial migration and vascular reconstruction. The concurrent progression of osteogenesis and angiogenesis is important for effective bone repair [9]. Current scaffold bioactivation strategies often rely on exogenous growth factors or peptides to enhance inductive potency, but high cost, limited stability, and potential immunological concerns continue to hinder translation [10]. In contrast, small molecules with favorable safety profiles, chemical stability, and bioactive potential may offer a more controllable and reproducible route for conferring durable biological function to scaffolds [11].

Photocuring-enabled 3D printing provides a high-precision and reproducible platform for fabricating defect-matched scaffolds with controllable macroscopic geometry and pore/channel architecture. Such architectural control can support cell anchorage, mass transport, tissue ingrowth, and early mechanical stabilization; however, geometry alone is insufficient to provide the biological cues required for coordinated bone and vascular regeneration [12]. We therefore selected photocrosslinkable SilMA as the structural matrix and incorporated L-leucine (L-Leu) as a small-molecule bioactive component, with the aim of integrating programmable physical support and biological regulation within a single scaffold.

L-Leu is an essential branched-chain amino acid involved in protein synthesis and metabolic regulation. Previous studies suggest that L-Leu may influence osteogenic differentiation, extracellular matrix deposition, and angiogenesis-related activity [13]. We therefore hypothesized that incorporating L-Leu into a photocrosslinkable SilMA scaffold would improve the cell–material microenvironment and enhance both osteogenic and angiogenic responses [14]. Because collagen maturation is essential for bone matrix formation, we further investigated whether PLOD2-associated collagen remodeling contributes to the osteogenic effects of SilMA@L-Leu [15].

**Fig. 1:**
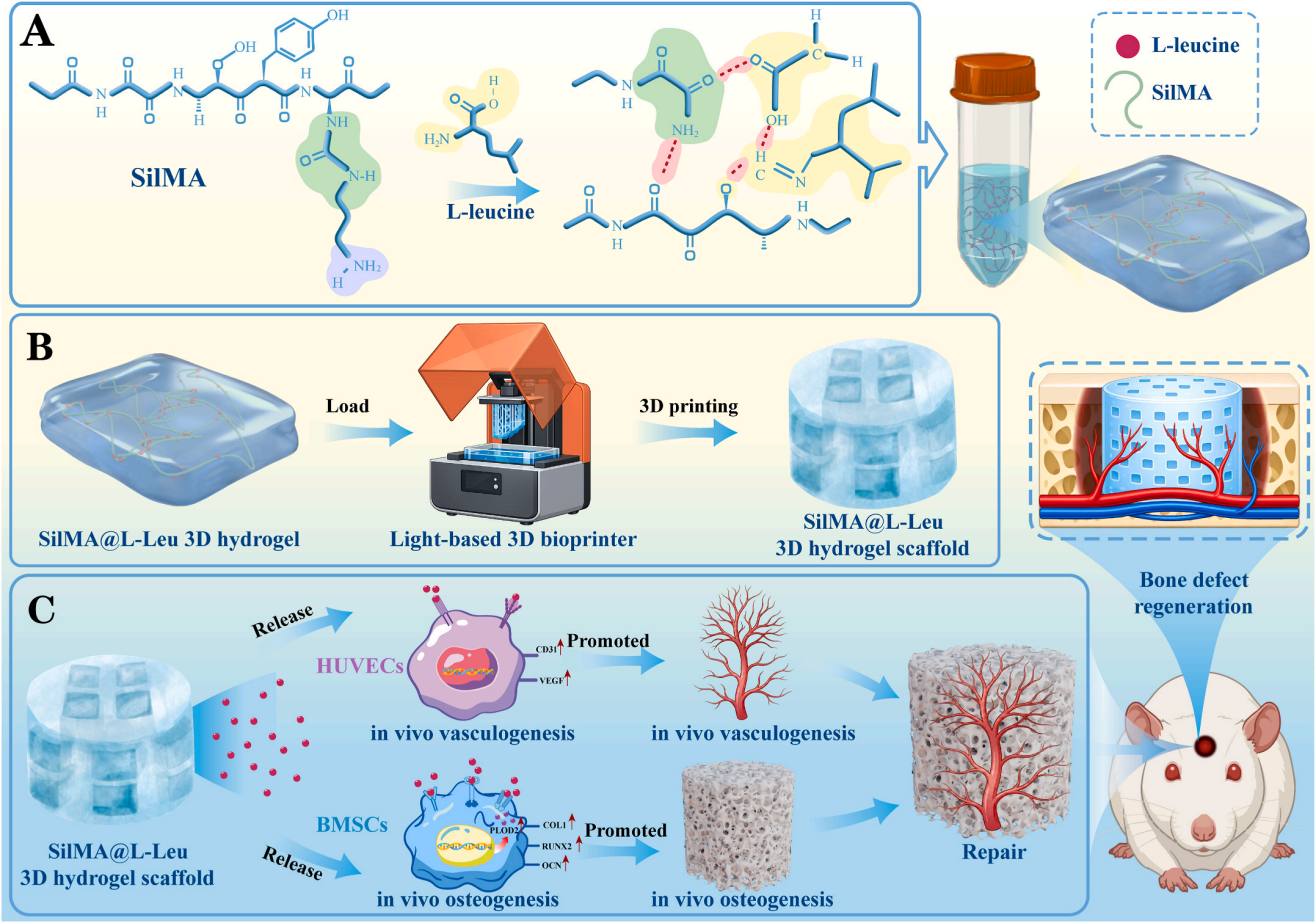
Schematic illustration of the design, fabrication, and mechanism of action of the 3D-printed SilMA@L-Leu hydrogel scaffold for osteogenic and angiogenic regulation and bone defect repair A) Preparation of the L-leucine (L-Leu)-functionalized methacrylated silk fibroin (SilMA) prepolymer solution. B) Fabrication of the 3D-printed SilMA@L-Leu hydrogel scaffold with programmable pore architecture via digital light processing (DLP)-based photopolymerization. C) Upon implantation, the scaffold promotes the migration and osteogenic differentiation of bone marrow-derived mesenchymal stem cells (BMSCs) as well as the migration and angiogenic tube formation of human umbilical vein endothelial cells (HUVECs), thereby supporting parallel osteogenic and angiogenic responses. D) The scaffold enhances the expression of osteogenesis-related (RUNX2, OCN) and angiogenesis-related (VEGFA, CD31) markers, leading to effective repair of critical-size calvarial defects. PLOD2-associated extracellular matrix (ECM) remodeling contributes to the pro-osteogenic effect of the scaffold. Created with Adobe Illustrator.

Accordingly, we fabricated and characterized 3D-printed SilMA@L-Leu scaffolds, evaluated their effects on BMSC osteogenic differentiation and HUVEC angiogenic behavior, and assessed their regenerative performance in a rat critical-size calvarial defect model. Proteomic screening and siRNA-mediated PLOD2 silencing were subsequently used to examine the potential involvement of PLOD2-associated collagen remodeling [16]. The overall experimental design is summarized in Fig. 1.

## 2 Experimental section

### 2.1 Fabrication of 3D-Printed SilMA@L-Leu hydrogel scaffolds

#### 2.1.1 Preparation of the L-Leu–loaded SilMA prepolymer solution

A photoinitiator-containing solvent was prepared by dissolving lithium phenyl-2,4,6-trimethylbenzoylphosphinate (LAP; Suzhou Yongqinquan Intelligent Equipment Co., Ltd., Suzhou, China) in phosphate-buffered saline (PBS, pH 7.4; KeyGen Biotech, Nanjing, China) to obtain a final concentration of 0.25% (w/v) (i.e., 2.5 mg mL^−1^). The solution was sterilized by filtration through a 0.22 μm membrane and stored protected from light until use. L-leucine (L-Leu; Shanghai Aladdin Biochemical Technology Co., Ltd., Shanghai, China) was prepared as a stock solution and diluted with the LAP/PBS solvent to yield three L-Leu working solutions with nominal concentrations of 100, 300, and 700 ng mL^−1^. Lyophilized methacrylated silk fibroin (SilMA) powder was then added to each working solution to a final concentration of 20% (w/v) and dissolved at 37 °C under light protection with gentle agitation for at least 3 h, until a homogeneous, transparent prepolymer solution without visible bubbles was obtained. The resulting prepolymer solutions were used for subsequent photocuring 3D printing.

#### 2.1.2 Photocuring 3D printing of SilMA@L-Leu scaffolds

A cylindrical porous scaffold model with an overall diameter of 5 mm and a height of 3 mm was constructed using computer-aided design (CAD). The CAD-defined macropore size within the scaffold was set to 800 μm. The model was imported into the slicing software of a digital light processing (DLP) 3D-printing system (BP8601 Pro, EFL, China) and sliced at a layer thickness of 100 μm. Accordingly, the 3-mm-high scaffold was divided into 30 layers.

The L-Leu-incorporated SilMA prepolymer solution was transferred into the resin vat and fabricated by layer-by-layer DLP photopolymerization. Each layer was exposed for 10 s at a light intensity of 8 mW cm^−2^. The first two layers served as base layers to improve adhesion to the build platform and were exposed under the same conditions. No additional support structures were required because the scaffold was printed directly on the build platform. A conventional linear printing speed was not applicable to this layer-wise DLP process because scaffold fabrication was primarily controlled by the predefined layer thickness and exposure time rather than by nozzle movement or laser scanning speed.

After printing, the scaffolds were carefully removed from the build platform. No additional post-printing light-curing treatment was performed. The printed scaffolds were sterilized by immersion in 75% (v/v) ethanol for 30 min, followed by thorough rinsing with sterile PBS to remove residual ethanol and uncured components. The sterilized scaffolds were stored at 4 °C protected from light until further use. The 800 μm value refers to the CAD-defined macroporous channels, whereas the smaller pores observed on the scaffold struts after lyophilization were evaluated separately by scanning electron microscopy (SEM) [17].

### 2.2 Characterization of 3D-Printed SilMA@L-Leu hydrogel scaffolds

#### 2.2.1 Scanning electron microscopy (SEM) of scaffold surface morphology

Four scaffold formulations were evaluated: SM (SilMA without L-Leu), SM@L100 (SilMA containing 100 ng mL^−1^ L-Leu), SM@L300 (SilMA containing 300 ng mL^−1^ L-Leu), and SM@L700 (SilMA containing 700 ng mL^−1^ L-Leu).

The microstructure of the 3D-printed SilMA@L-Leu hydrogel scaffolds (SilMA and SilMA@L-Leu containing 100, 300, or 700 ng mL^−1^ L-Leu) was examined by FE-SEM (SU8010, Hitachi, Japan). After printing and washing, the scaffolds were frozen at −80 °C overnight, lyophilized to constant weight (FD-1A-50, Beijing Boyikang, China), mounted on conductive carbon tape, sputter-coated with gold, and imaged at 5 kV. Representative images were acquired from both the scaffold surface and strut regions. Pore parameters were analyzed using ImageJ (National Institutes of Health, USA). Pore-size quantification focused on the micropores formed on the strut surfaces after lyophilization, rather than the CAD-defined macroporous channels. Multiple fields per group were randomly selected at the same magnification, and the mean micropore size was calculated for intergroup comparison.

#### 2.2.2 Determination of porosity

The porosity of the scaffolds was determined using an ethanol displacement (ethanol uptake) method. Lyophilized samples were first weighed to obtain the dry mass (*W*_*h*_). Each sample was then completely immersed in absolute ethanol and reweighed after removal to obtain the ethanol-saturated mass (*W*_*a*_). Porosity was calculated according to:

$$Porosity\left(\%\right)=\frac{W_{a}‐W_{h}}{\rho_{\text{ethanol}}V}\times 100\%$$

where ρethanol is the density of ethanol and *V* is the scaffold (hydrogel) volume.

#### 2.2.3 Compressive mechanical testing

The compressive properties of the hydrogel scaffolds were assessed by uniaxial compression testing on a universal testing machine (ZwickRoell, Germany). Residual surface liquid was removed before testing. Samples were placed between parallel platens and compressed at a constant rate, while force–displacement data were recorded and converted to engineering stress–strain curves. The compressive modulus was determined from the slope of the initial linear region. Three independent samples were analyzed for each group (n = 3).

#### 2.2.4 Swelling behavior

The swelling behavior of the hydrogel scaffolds was assessed using lyophilized samples from four groups (n = 3). The scaffolds were frozen at −80 °*C* overnight and lyophilized under vacuum to constant weight to obtain fully dehydrated specimens. The dry weight was recorded as *Wb*. Each scaffold was then immersed in 1 mL phosphate-buffered saline (PBS). At predetermined time points, samples were removed, gently blotted to remove surface-adhered liquid, and immediately weighed to obtain the wet weight (*Ww*). Changes in Ww over time were used to evaluate swelling kinetics, and the swelling ratio was calculated as:

$$Swellingratio\left(\%\right)=\frac{W_{w}‐W_{b}}{W_{b}}\times 100\%$$

All measurements were conducted at room temperature.

#### 2.2.5 In vitro degradation

To assess mass loss under in vitro conditions, scaffolds from four groups (n = 3) were pre-frozen at −80 °C overnight and lyophilized under vacuum until a constant mass was reached. The initial dry mass was recorded as *D*_*0*_. Each scaffold was then incubated in 1 mL PBS at 37 °C to mimic physiological conditions. At predetermined time points, samples were retrieved and thoroughly rinsed with deionized water to remove residual salts. The scaffolds were subsequently dried to a constant mass and reweighed to obtain the remaining dry mass (*D*_*w*_). The extent of degradation was quantified as the remaining mass ratio:

$$Remainingmass\left(\%\right)=\frac{D_{w}}{D_{0}}\times 100\%$$

#### 2.2.6 In vitro release of L-leucine

SM@L100, SM@L300, and SM@L700 scaffolds were individually immersed in 1 mL of PBS (pH 7.4) and incubated at 37 °C (n = 3). At days 1, 3, 7, 14, 21, and 28, the entire release medium was collected and replaced with an equal volume of fresh PBS. L-Leu concentrations were determined using a competitive ELISA kit according to the manufacturer's instructions. Absorbance was measured at 450 nm, and concentrations were calculated from a four-parameter logistic standard curve. The cumulative amount released at the nth time point was calculated as:

$$M_{n}=\sum_{i=1}^{n}C_{i}V$$

The percentage of L-Leu retained in the scaffold was calculated as:

$$L‐Leuretention\left(\%\right)=\frac{M_{0}‐M_{n}}{M_{0}}\times 100\%$$

#### 2.2.7 pH evaluation of the immersion medium

SM, SM@L100, SM@L300, and SM@L700 scaffolds were individually immersed in 1 mL of PBS (pH 7.4) and incubated at 37 °C. Three independent samples were analyzed for each group (n = 3). The pH of the immersion medium was measured using a calibrated pH meter on days 0, 1, 3, 7, 14, 21, 28, 35, 42, 49, 56, and 63. The pH meter was calibrated with standard buffer solutions before each measurement. Data are presented as mean ± standard deviation.

#### 2.2.8 Determination of L-Leu encapsulation efficiency

Printed and washed SM@L100, SM@L300, and SM@L700 scaffolds were lyophilized, cut into small pieces, immersed in 1 mL PBS, sonicated for 30 min, and incubated at 37 °C for 24 h (n = 3). After centrifugation at 3000 rpm for 10 min, the supernatant was collected. Extraction was repeated until L-Leu was below the detection limit. L-Leu concentrations were measured using a competitive ELISA kit. Absorbance was recorded at 450 nm, and concentrations were calculated from a four-parameter logistic standard curve with dilution correction.

$$M_{\text{encapsulated}}=\sum_{i=1}^{n}C_{i}V_{i}$$

$$Encapsulation\;efficiency\left(\%\right)=\frac{M_{encapsulated}}{M_{initial}}\times 100\%$$

where C_i_ and V_i_ are the concentration and volume of each extract, respectively, and M_initial_ is the theoretical amount of L-Leu initially added. Data are presented as mean ± SD.

#### 2.2.9 Water contact angle measurement

Surface wettability was assessed by water contact-angle measurement. Lyophilized scaffolds from four groups (n = 3) were tested using a contact-angle goniometer (Powereach, Lianke Instruments, China). A 5 μL droplet of deionized water was deposited on the scaffold surface, and the contact angle was determined after droplet stabilization using the instrument software. Three measurements were performed at different positions on each sample, and the average value was used for analysis. All tests were conducted at room temperature.

#### 2.2.10 Fourier transform infrared (FTIR) spectroscopy

FTIR spectra of lyophilized scaffold samples were collected using a Fourier transform infrared spectrometer (Thermo Fisher Scientific, USA) over the wavenumber range of 4000–500 cm^−1^ at a resolution of 4 cm^−1^, with at least 32 scans per sample. Before analysis, the dried samples were ground into fine powders. The spectra were baseline-corrected and normalized for intergroup comparison.

#### 2.2.11 Rheological measurements

Rheological measurements were performed using a rotational rheometer (Discovery HR-2, TA Instruments, USA) with a parallel-plate geometry at 25 °C. The apparent viscosity of the uncured precursor solutions was measured over a shear-rate range of 1–20 s^−1^. Photocuring kinetics were evaluated by a time sweep under 405-nm irradiation at 30 mW cm^−2^ for 30 s, while the storage modulus (G′) and loss modulus (G″) were recorded. After photocuring, strain sweeps were conducted from 0.1% to 100% at 1 rad s^−1^, followed by frequency sweeps from 0.1 to 100 rad s^−1^ at 1% strain. All measurements were performed in triplicate.

#### 2.2.12 Evaluation of hydrogel network stability

Compression testing and rheological analysis were used to indirectly evaluate the network stability and effective network constraints of the SilMA@L-Leu hydrogels. Fully hydrated SM, SM@L100, SM@L300, and SM@L700 scaffolds were subjected to uniaxial compression testing. Stress–strain curves were recorded, and the compressive modulus was calculated from the slope of the initial linear region.Rheological measurements were performed using a rotational rheometer at 25 °C. A strain sweep from 0.1% to 100% was first conducted at a fixed angular frequency of 1 rad s^−1^ to determine the linear viscoelastic region. Subsequently, a frequency sweep from 0.1 to 100 rad s^−1^ was performed at 1% strain, and the storage modulus (*G′*) and loss modulus (*G″*) were recorded. Changes in hydrogel elasticity, structural stability, and effective network constraints after L-Leu incorporation were evaluated by comparing the compressive modulus, *G′*, and *G″* among groups.

### 2.3 Cell culture and maintenance

#### 2.3.1 Culture of bone Marrow–Derived mesenchymal stem cells (BMSCs)

Primary BMSCs were isolated from 4–6-week-old male Sprague–Dawley rats (Nanjing Qinglongshan, Nanjing, China). Bone marrow was flushed from the femora and tibiae with low-glucose DMEM (KeyGen Biotech, Nanjing, China) containing 1% penicillin–streptomycin, filtered through a 200-mesh strainer, centrifuged, and resuspended in complete medium supplemented with 10% fetal bovine serum. Cells were cultured at 37 °C in a humidified atmosphere containing 5% CO_2_, with medium replacement after 24 h and every 2–3 days thereafter. Cells at passages 2–5 were used for all experiments.

#### 2.3.2 Culture of human umbilical vein endothelial cells (HUVECs)

HUVECs were purchased from Haixing Biosciences (Suzhou, China) and cultured in high-glucose DMEM supplemented with 10% fetal bovine serum and 1% penicillin–streptomycin (all reagents from KeyGen Biotech, Nanjing, China) at 37 °C in a humidified atmosphere containing 5% CO_2_. Cells were routinely passaged, and those in the logarithmic growth phase were used for subsequent experiments.

### 2.4 Cell viability and proliferation

#### 2.4.1 Preparation of hydrogel extracts (conditioned media)

Scaffold extracts were prepared by immersing hydrogel scaffolds from each group (5 mm × 3 mm) in 1.5 mL of the corresponding culture medium at 37 °C for 24 h. The supernatants were collected after centrifugation, filtered through a 0.22 μm membrane, and used immediately or stored at 4 °C for up to 24 h before use.

#### 2.4.2 CCK-8 assay

Cell proliferation was assessed using a Cell Counting Kit-8 (CCK-8; Baisha Biotechnology, China). BMSCs and HUVECs were seeded in 96-well plates at densities of 3 × 10^3^ and 5 × 10^3^ cells per well, respectively (n = 3), and cultured with the corresponding hydrogel extracts after attachment. On days 1, 3, and 5, the culture medium was replaced with fresh medium containing 10% (v/v) CCK-8 reagent; serum-free medium was used for HUVECs. After incubation at 37 °C for 1 h, absorbance at 450 nm was measured using a microplate reader (800 TS, Thermo Scientific, USA).

### 2.5 Live/dead staining

Cell viability was evaluated by Live/Dead staining in a 24-well Transwell-based indirect co-culture system. BMSCs or HUVECs were seeded in the lower chambers, and the corresponding hydrogel scaffolds were placed in the upper inserts (6.5 mm diameter, 8.0 μm pore size; Jet Biofil, China) to establish a non-contact co-culture system (n = 3). Each well contained 1 mL of complete medium, and the cultures were maintained at 37 °C in a humidified atmosphere with 5% CO_2_ for 1, 3, or 5 days. At each time point, cell viability was assessed using a Calcein-AM/propidium iodide (PI) Live/Dead staining kit (Beyotime Biotechnology, China). After removal of the culture medium, the staining solution was added and incubated at 37 °C for 30 min in the dark. The cells were then gently rinsed with PBS, and fluorescence images were acquired using a Leica THUNDER imaging system (THUNDER Imager, Leica Microsystems, Germany).

### 2.6 ALP staining

BMSCs were cultured in a Transwell-based indirect co-culture system with the hydrogel scaffolds placed in the upper inserts and osteogenic differentiation induced using osteogenic medium (PronoSci, China), which was refreshed every 2 days. On days 7 and 14, alkaline phosphatase (ALP) staining was performed on three parallel samples per group (n = 3) using a BCIP/NBT ALP staining kit (Beyotime Biotechnology, China). After fixation, the cells were incubated with the staining solution in the dark until color development, rinsed with PBS, and imaged under a microscope.

### 2.7 Alizarin Red S staining

Extracellular matrix mineralization was evaluated by Alizarin Red S staining in a Transwell-based indirect co-culture system. BMSCs were cultured with hydrogel scaffolds under osteogenic induction (PronoSci, China) for 14 or 21 days, with medium renewal every 2 days. After fixation, the cells were stained with Alizarin Red S (Solarbio, China), rinsed with PBS, and imaged under a light microscope (n = 3).

### 2.8 Tube formation assay

HUVEC tube formation was evaluated in a Transwell-based indirect co-culture system. Matrigel (MCE, China) was added to 24-well plates (50 μL per well) and gelled at 37 °C for 30 min, after which HUVECs were seeded at 1 × 10^4 cells per well. Hydrogel scaffolds were placed in the upper inserts for non-contact co-culture (n = 3). The cells were incubated at 37 °C in a humidified atmosphere containing 5% CO_2_, and images were acquired at 6 and 8 h using a Leica THUNDER imaging system (Leica Microsystems, Germany). Tube formation was quantified by ImageJ in terms of total tube length, node number, and branch number.

### 2.9 Immunofluorescence staining

Immunofluorescence staining was performed to evaluate osteogenesis- and angiogenesis-related protein expression in cells cultured in Transwell-based indirect co-culture systems. For BMSCs under osteogenic induction, cells were collected on days 7 and 21 and incubated with primary antibodies against COL1, RUNX2, and OCN (Affinity Biosciences, China). For HUVECs, cells were collected on day 3 and incubated with primary antibodies against CD31 and VEGF (Affinity Biosciences, China). To assess key pathway-associated proteins, BMSCs were additionally collected on day 7 and stained for COL1, PLOD2, and LOX. In all cases, cells were fixed with 4% paraformaldehyde, permeabilized with 0.1% Triton X-100, and blocked with a commercial immunofluorescence blocking solution (Yamei, China) according to the manufacturer's instructions. Samples were then incubated with the corresponding primary antibodies overnight at 4 °C, followed by fluorophore-conjugated secondary antibodies for 1 h at room temperature in the dark and DAPI counterstaining. Fluorescence images were acquired using a Leica THUNDER imaging system (Leica Microsystems, Germany), whereas confocal images for COL1/PLOD2/LOX staining were obtained using an Olympus confocal microscope (Olympus, Japan). Fluorescence intensity was quantified with ImageJ where indicated (n = 3).

### 2.10 Transwell migration assay

Cell migration was evaluated using a Transwell assay. BMSCs or HUVECs were resuspended in serum-free medium and seeded into the upper chambers at a density of 2 × 10^4 cells per well. The lower chambers were filled with hydrogel extracts from each group supplemented with 10% FBS to establish a chemotactic gradient. The cells were incubated at 37 °C in a humidified atmosphere containing 5% CO_2_, and migrated cells were evaluated at 24 and 48 h for BMSCs or at 12 and 24 h for HUVECs. After incubation, non-migrated cells on the upper membrane surface were removed with a cotton swab. Cells that migrated to the underside of the membrane were fixed with 4% paraformaldehyde for 15 min, stained with 0.1% crystal violet (Solarbio, Beijing, China) for 20 min, rinsed with PBS, and imaged using a Leica THUNDER imaging system (THUNDER Imager, Leica Microsystems, Germany). Migrated cells were quantified using ImageJ (National Institutes of Health, USA) by cell counting.

### 2.11 In vitro scratch (wound-healing) assay

Cell migration was further evaluated using an in vitro scratch (wound-healing) assay in an indirect co-culture system. BMSCs or HUVECs were seeded in 6-well plates and cultured to 80–90% confluence. A linear scratch was created across the cell monolayer using a sterile 200 μL pipette tip, and detached cells were removed by gentle PBS washing. Serum-free medium was then added, and Transwell inserts loaded with hydrogel scaffolds from each group were placed above the wells to establish a non-contact co-culture condition (n = 3). Images of the same wound region were acquired at 0 and 24 h using a Leica THUNDER imaging system (THUNDER Imager, Leica Microsystems, Germany). Wound closure was quantified using ImageJ by measuring the change in wound area, and the wound closure rate was calculated.

### 2.12 siRNA-mediated gene silencing

To investigate the role of the candidate gene in regulating BMSC osteogenic differentiation, we used small interfering RNA (siRNA) to knock down the target gene. Three siRNA sequences were designed and commercially synthesized (Sangon Biotech, Shanghai, China; see Table 1). Functional validation was then performed to assess their effect on BMSC osteogenic activity.

**Table 1:** Sequences of siRNAs targeting PLOD2.

Target gene	siRNA ID	Sense strand (5' → 3′)	Antisense strand (5' → 3′)
PLOD2	siPLOD2-316	GGUUGUCAUGUUUACUGAAdTdT	UUCAGUAAACAUGACAACCdTdT
PLOD2	siPLOD2-261	AGAAAGUGAGAUUAAUGAAdTdT	UUCAUUAAUCUCACUUUCUdTdT
PLOD2	siPLOD2-771	CCAAGAUUCUCCUGAAUUAdTdT	UAAUUCAGGAGAAUCUUGGdTdT

### 2.13 Quantitative real-time PCR analysis

RT–qPCR was performed to evaluate gene expression in cells cultured in Transwell-based indirect co-culture systems. For osteogenic analysis, BMSCs were cultured with hydrogel scaffolds in osteogenic medium for 10–14 days, whereas HUVECs were co-cultured with hydrogel scaffolds in complete medium for 3 days to assess angiogenesis-related gene expression. At the indicated time points, total RNA was extracted using an RNA isolation kit (KeyGen Biotech, Nanjing, China), and RNA concentration and purity were determined using a microvolume spectrophotometer (Bio-Rad, USA). cDNA was synthesized using a reverse transcription kit (TransGen Biotech, China), followed by RT–qPCR analysis (Bio-Rad, USA). The expression levels of osteogenesis-related genes (RUNX2, COL1A1, and OCN) in BMSCs and angiogenesis-related genes (CD31 and VEGFA) in HUVECs were quantified. GAPDH was used as the internal reference, and relative gene expression was calculated using the 2−ΔΔCt method. Primer sequences are listed in Table 2.

**Table 2:** **Primer sequences used for RT–qPCR** Primer sequences of genes related to osteogenesis, angiogenesis, and extracellular matrix remodeling.

Genes	5′-3′	Primers
GAPDH	Forward	GACATGCCGCCTGGAGAAAC
	Reverse	AGCCCAGGATGCCCTTTAGT
RUNX2	Forward	TCTGACCGCCTCAGTGATTT
	Reverse	GTGTCTGCCTGGGATCTGTA
COL1A1	Forward	CCCTGCTGGAGAAGAAGGAA
	Reverse	AGGAGAACCTTTGGGACCAG
OCN	Forward	GACCCTCTCTCTGCTCACTC
	Reverse	GGGCTCCAAGTCCATTGTTG
CD31	Forward	AACAGTGTTGACATGAAGAGCC
	Reverse	TGTAAAACAGCACGTCATCCTT
VEGFA	Forward	AGGGCAGAATCATCACGAAGT
	Reverse	AGGGTCTCGATTGGATGGCA
HIF-1α	Forward	TGACCACTGCTAAGGCATCA
	Reverse	GGCTCCTTGGATGAGCTTTG
PLOD2	Forward	AGTGAAGTACTCGCCCGAAA
	Reverse	ATTTGCATCCACCTCCCTGA
LOX	Forward	GCTTATGTCTGGAGGACACT
	Reverse	TAACATCCGGGACTCAACCC
ALP	Forward	GTGTCGGAAGATGGGAAAGC
	Reverse	ACTGGTTCGGCCTGGAATTA
BMP2	Forward	GGACATCCACTCCACAAACG
	Reverse	GCTGGACTTAAGACGCTTCC

For mechanistic analysis, siPLOD2-transfected BMSCs were cultured in the same indirect co-culture system under osteogenic induction. On day 7 or the indicated endpoint, total RNA was extracted, reverse-transcribed, and analyzed by RT–qPCR as described above. The expression of pathway-related genes, including HIF-1α, PLOD2, and LOX, as well as osteogenesis-related genes, including ALP, RUNX2, and BMP2, was quantified using GAPDH as the internal reference. Relative gene expression was calculated using the 2−ΔΔCt method. Primer sequences are listed in Table 2.

### 2.14 Western blotting

Total protein was extracted using lysis buffer (Biosharp Life Sciences, Anhui, China), and protein concentration was determined using a BCA assay kit (Beyotime, China). Equal amounts of protein were separated by SDS–PAGE and transferred onto poly(vinylidene fluoride) (PVDF) membranes. The membranes were blocked with 3% bovine serum albumin for 2 h at room temperature and then incubated overnight at 4 °C with primary antibodies against osteogenesis-related proteins (COL1, OCN, and RUNX2), angiogenesis-related proteins (VEGF and CD31), and loading controls (GAPDH and TUBULIN) (Affinity Biosciences, China). After washing with TBST, the membranes were incubated with HRP-conjugated secondary antibodies at room temperature. Protein bands were visualized using an enhanced chemiluminescence substrate on a Bio-Rad imaging system, and band intensities were quantified using ImageJ.

### 2.15 Proteomics analysis

For proteomic analysis, BMSCs cultured without exposure to any scaffold or material served as the Control group, whereas BMSCs indirectly co-cultured with SM@L300 scaffolds constituted the SM@L300 group. Osteogenically induced samples were lysed in 350 μL of lysis buffer (Omicsolution) and sonicated in an ice bath for 20 min. After centrifugation (4 °C, 12,000 rpm, 10 min), the supernatants were collected, and protein concentration was determined by BCA assay (Beyotime). For proteomic analysis, 30 μg of protein per sample was subjected to reduction, alkylation, and tryptic digestion. The resulting peptides were desalted by solid-phase extraction, vacuum-dried, and reconstituted in mobile phase A. LC–MS/MS analysis was performed on a Vanquish Neo UHPLC system coupled to an Orbitrap Astral mass spectrometer (Thermo Scientific) with an injection amount of 500 ng. Peptides were separated on an EASY-Spray™ column (150 μm × 15 cm) using a 6.9 min gradient with 0.1% formic acid in water as mobile phase A and 0.1% formic acid in 80% acetonitrile as mobile phase B. Data were acquired in DIA mode (MS1: *m*/*z* 380–980, resolution 240k; MS2: *m*/*z* 150–2000; NCE 25%). Raw data were analyzed against the UniProt rat database using DIA-NN (v1.8.1), with precursor and fragment mass tolerances set to 20 ppm, up to two missed cleavages allowed, carbamidomethylation of cysteine as a fixed modification, and N-terminal acetylation and methionine oxidation as variable modifications. The false discovery rate was controlled at <1%. Differentially expressed proteins between the SM@L300 and Control groups were screened according to the criteria used in the proteomic analysis. The identified proteins were subsequently analyzed by hierarchical clustering, subcellular localization, Kyoto Encyclopedia of Genes and Genomes (KEGG) classification, Gene Ontology (GO) enrichment, COG/KOG functional classification, pathway–protein association analysis, and protein–protein interaction network analysis.

### 2.16 Animal model

Adult male rats (6–8 weeks old, 200–300 g; total n = 18) were randomly assigned to three groups: Control, SM@L300, and Allograft. All animals underwent creation of a critical-size calvarial defect. The Control group received no implant after defect creation, the SM@L300 group received an SM@L300 scaffold, and the Allograft group received an allogeneic bone graft.The allogeneic grafts were prepared from femoral cancellous bone harvested from healthy donor rats of the same batch and age and were sterilized by autoclaving before implantation.

Anesthesia was induced by intraperitoneal injection of 5% sodium pentobarbital (0.3 mL per rat). After routine shaving and disinfection, a midline incision (≈1.5–2.0 cm) was made over the calvarium, and the parietal bone was exposed by blunt dissection. A standardized full-thickness calvarial defect (4–5 mm in diameter) was created using a dental trephine under continuous saline irrigation, while preserving the integrity of the dura mater. The corresponding material was implanted according to group assignment, whereas the Control group received no implant. The periosteum and skin were closed in layers with 7-0 and 4-0 sutures, respectively. During postoperative recovery, the animals were kept warm until full awakening and received penicillin for infection prophylaxis.

### 2.17 Micro-CT evaluation

Calvarial specimens were fixed in 4% paraformaldehyde, mounted on the sample stage, and scanned using a high-resolution micro-computed tomography system (VNC-102, Pingseng Healthcare, China). Scans were acquired in continuous mode with a radial field of view of 45 mm and a maximum axial range of 86 mm. The reconstructed voxel size was 6 μm, and the spatial resolution was better than 4 μm at 10% MTF. After three-dimensional reconstruction using Recon software, the datasets were imported into Avatar software (Pingseng Healthcare, China) for post-processing and quantitative analysis. Bone volume fraction (BV/TV), bone mineral density (BMD), trabecular thickness (Tb.Th), and trabecular number (Tb.N) were quantified to evaluate bone regeneration.

### 2.18 Gross observation and histological evaluation

Following micro-CT analysis, the fixed specimens were decalcified in 10% EDTA, dehydrated through a graded ethanol series, embedded in paraffin, and serially sectioned at 7 μm. The sections were stained with H&E, Masson's trichrome, and Goldner's trichrome (Solarbio, China) to evaluate tissue morphology, bone matrix mineralization, and collagen deposition within the defect area. To further assess osteogenic activity and vascularization, immunohistochemical staining for COL1 and double immunofluorescence staining for COL1 and CD31 were performed using primary antibodies from Affinity Biosciences, USA. The stained sections were examined under a microscope, and representative images were acquired for qualitative and quantitative analysis.

### 2.19 Preliminary histological safety assessment of major organs

For a preliminary histological safety assessment, the heart, liver, spleen, lung, and kidney were harvested at 6 and 12 weeks postoperatively. The tissues were fixed in 4% paraformaldehyde, processed through routine dehydration, embedded in paraffin, and sectioned. Hematoxylin and eosin (H&E) staining was performed according to standard histological procedures. Representative sections were examined under a light microscope to identify overt histopathological abnormalities, including tissue disorganization, necrosis, or marked inflammatory infiltration. This assessment was limited to morphological observations and was not intended to provide a comprehensive evaluation of systemic toxicity.

### 2.20 Statistical analysis

All quantitative data are presented as mean ± standard deviation (SD). Unless otherwise specified, n denotes the number of independent experimental units included in the statistical analysis. For material characterization, n represents independently prepared scaffold specimens. For in vitro assays, n represents independent experimental repeats. Technical replicates, including replicate wells, repeated measurements, or multiple microscopic fields obtained from the same experimental sample, were averaged to generate a single value for each independent replicate and were not treated as independent observations. For the animal experiments, each animal was considered an independent biological replicate and the experimental unit. The exact sample size is provided in the corresponding Methods subsection or figure legend.

Statistical analyses were performed using GraphPad Prism 10. Differences among multiple groups at a single time point were analyzed using one-way analysis of variance (ANOVA) followed by Tukey's multiple-comparisons test. Data involving treatment and time were analyzed using two-way ANOVA followed by Tukey's multiple-comparisons test. Repeated-measures two-way ANOVA was used only when measurements were repeatedly obtained from the same independent samples over time. Comparisons between two independent groups were performed using an unpaired two-tailed Student's t-test. A value of p < 0.05 was considered statistically significant. Significance was indicated as *p < 0.05, **p < 0.01, ***p < 0.001, and ****p < 0.0001; ns, not significant.

## 3 Results

### 3.1 Fabrication of 3D-Printed SilMA@L-Leu hydrogel scaffolds

SilMA hydrogel scaffolds incorporating L-leucine (L-Leu) were successfully fabricated by combining photoinduced free-radical polymerization with photocurable 3D printing. LAP was first dissolved in PBS (pH 7.4), followed by the addition of L-Leu at 100, 300, or 700 ng mL^−1^. All formulations remained clear and homogeneous, without visible precipitation or phase separation, indicating good compatibility of L-Leu within the precursor system. After incorporation of lyophilized SilMA (20% w/v) and dissolution at 37 °C under light protection, the resulting prepolymer solutions were transparent and bubble-free, consistent with the rheological and compositional uniformity required for stable photocuring and printing.

**Fig. 2:**
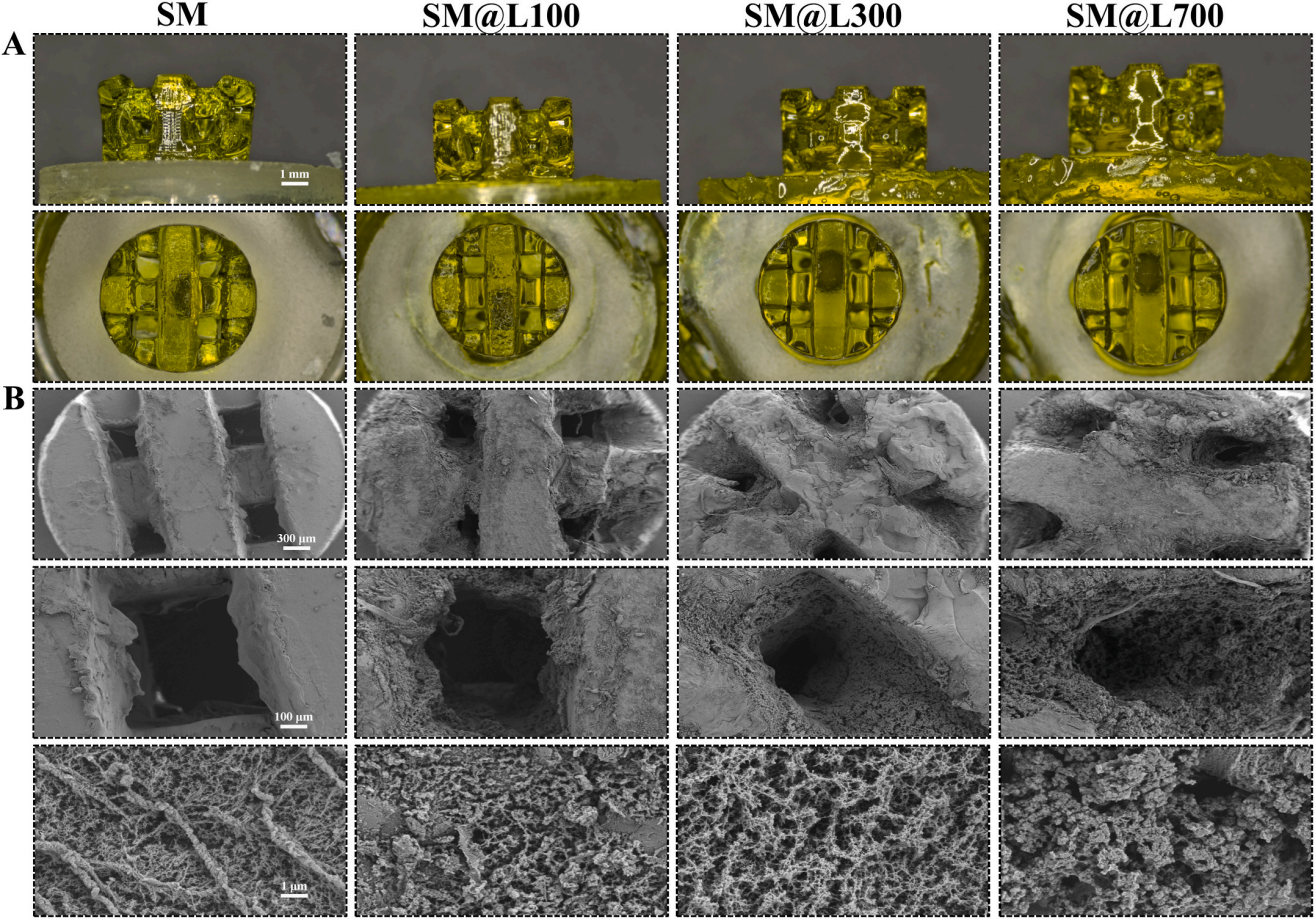
Fabrication and structural characterization of 3D-printed SilMA@L-Leu hydrogel scaffolds. A) Representative photographs of 3D-printed cylindrical scaffolds of SilMA (SM) and SilMA containing L-Leu at concentrations of 100 ng mL-1 (SM@L100), 300 ng mL-1 (SM@L300), and 700 ng mL-1 (SM@L700), demonstrating well-defined geometry and shape fidelity. B) Scanning electron microscopy (SEM) images showing the porous microstructure of the scaffold struts, with interconnected pores and intact architecture across all groups. SM, SilMA; SM@L100, SilMA@L-Leu-100; SM@L300, SilMA@L-Leu-300; SM@L700, SilMA@L-Leu-700.

The precursor solutions were then processed according to the CAD-designed model to generate cylindrical porous scaffolds with a diameter of ∼5 mm, a height of ∼3 mm, and regularly aligned internal channels with a designed pore size of ∼800 μm. Under a light intensity of 8 mW cm^−2^ and an exposure time of 10 s per layer, all formulations showed stable layer-by-layer curing and yielded scaffolds with well-preserved geometry, clear boundaries, and no evident collapse or channel occlusion. Following ethanol sterilization and PBS rinsing, the printed constructs maintained their predefined three-dimensional architecture and structural integrity during post-processing and storage. These results indicate that L-Leu incorporation did not compromise precursor stability, printability, or scaffold fidelity (Fig. 2A).

### 3.2 Material characterization of 3D-Printed SilMA@L-Leu hydrogel scaffolds

**Fig. 3:**
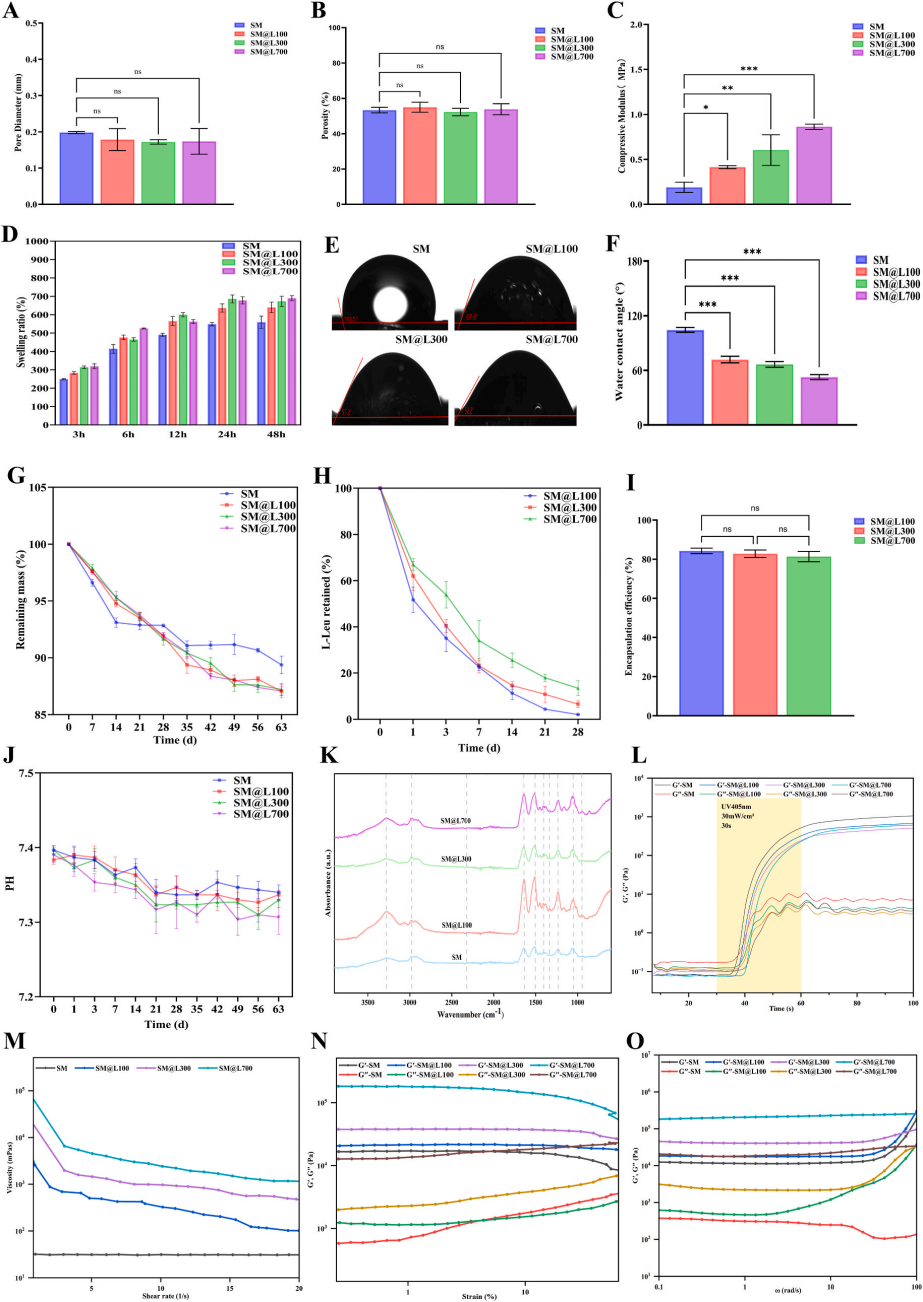
Physicochemical, mechanical, release, and rheological characterization of 3D-printed SilMA@L-Leu hydrogel scaffolds. (A) Micropore size. (B) Porosity. (C) Compressive modulus. (D) Swelling in PBS over 48 h. (E,F) Representative water droplets and contact-angle quantification. (G) Remaining mass during 63-day degradation in PBS. (H) L-Leu retention over 28 days. (I) L-Leu encapsulation efficiency. (J) Changes in the pH of the immersion medium over 63 days. (K) FTIR spectra. (L) Photorheological time sweep during 405-nm irradiation. (M) Apparent viscosity as a function of shear rate. (N) Strain sweep. (O) Frequency sweep. SM, SilMA; SM@L100, SM@L300, and SM@L700, SilMA scaffolds containing 100, 300, and 700 ng mL^−1^ L-Leu, respectively. Data are presented as mean ± SD (n = 3 independently prepared scaffold specimens). *p < 0.05, **p < 0.01, and ***p < 0.001; ns, not significant.

SEM imaging showed that all scaffolds retained an interconnected porous morphology with no obvious structural collapse after L-Leu incorporation (Fig. 2B). Consistently, quantitative analysis revealed comparable pore sizes across groups, with average values of 0.20 ± 0.01 mm for pure SilMA and 0.18 ± 0.03, 0.17 ± 0.01, and 0.18 ± 0.04 mm for the SM@L100, SM@L300, and SM@L700 groups, respectively, without significant differences (Fig. 3A). These values correspond to the microscale pores within the lyophilized scaffold struts observed by SEM, rather than the CAD-defined macroporous channels (∼800 μm), indicating that L-Leu loading did not perturb scaffold microarchitecture.

Porosity measurements further supported the structural consistency of the printed scaffolds (Fig. 3B). All groups showed comparable porosity values within a narrow range of 50%–55%, with no significant differences among formulations. Specifically, the porosity was 52% ± 3% for pure SilMA and 54% ± 4%, 51% ± 3%, and 53% ± 4% for the SM@L100, SM@L300, and SM@L700 groups, respectively. No concentration-dependent trend was observed with increasing L-Leu content. Together with the pore-size analysis, these results indicate that L-Leu incorporation did not perturb the porous architecture of the SilMA scaffolds, thereby providing a structurally comparable basis for subsequent biological evaluation.

With micropore diameter and porosity remaining comparable among the four formulations, we next evaluated the mechanical, hydration, release, chemical, and rheological properties of the scaffolds. Uniaxial compression testing showed that the compressive modulus was significantly higher in all L-Leu-containing groups than in the SM group (Fig. 3C). The modulus increased numerically from approximately 0.2 MPa in SM to approximately 0.4, 0.6, and 0.85 MPa in SM@L100, SM@L300, and SM@L700, respectively, indicating that L-Leu incorporation improved the compressive performance of the SilMA scaffolds.

All formulations exhibited rapid water uptake during the initial immersion period and gradually approached swelling equilibrium at 24–48 h (Fig. 3D). The L-Leu-containing groups showed numerically higher swelling ratios than the SM group, particularly at the later time points. Water contact-angle measurements further demonstrated that L-Leu incorporation significantly improved scaffold surface wettability (Fig. 3E and F). The mean contact angle decreased from approximately 103.5° in the SM group to approximately 67.6°, 57.2°, and 52.2° in the SM@L100, SM@L300, and SM@L700 groups, respectively.

During the 63-day in vitro degradation test, all scaffolds showed slow and gradual mass loss (Fig. 3G). Approximately 87%–89% of the initial mass remained at day 63, and the mass-loss profiles were broadly comparable among groups. These results indicate that L-Leu incorporation did not cause rapid scaffold disintegration or markedly alter the overall degradation behavior.

The percentage of L-Leu retained in the scaffolds decreased rapidly during the initial 1–3 days and subsequently declined more gradually through day 28 (Fig. 3H), indicating an initial burst-release phase followed by a slower release stage. At later time points, the SM@L700 formulation retained a higher proportion of L-Leu than the lower-loading formulations. The encapsulation efficiencies of SM@L100, SM@L300, and SM@L700 were approximately 81%–84%, with no significant differences among the three groups (Fig. 3I). In addition, the pH of the immersion medium remained within a narrow near-neutral range throughout the 63-day incubation period, with no marked formulation-dependent acidification or alkalinization (Fig. 3J).

Although the three L-Leu-loaded formulations showed comparable encapsulation efficiencies, all formulations exhibited an initial rapid-release phase followed by continued retention and a slower release stage during the 28-day observation period. The suitability of SM@L300 was subsequently evaluated by integrating its release characteristics with its biological performance.

FTIR spectra of all formulations showed the characteristic absorption bands of the SilMA matrix (Fig. 3K). The broad band at approximately 3200–3400 cm^−1^ was attributed to O–H and N–H stretching vibrations, while the bands near 1640 and 1500–1540 cm^−1^ corresponded mainly to amide I C=O stretching and amide II N–H bending/C–N stretching, respectively. After L-Leu incorporation, the principal peak positions remained largely unchanged, and no distinct new absorption bands were observed, indicating that L-Leu loading did not induce detectable new covalent-bond formation or disrupt the primary chemical framework of SilMA. Minor changes in peak shape and intensity may reflect alterations in the local intermolecular environment.

In situ photorheological analysis showed that both the storage modulus (G′) and loss modulus (G″) increased rapidly after initiation of 405-nm irradiation, confirming rapid photocrosslinking of all formulations (Fig. 3L). Following irradiation, G′ exceeded G″ and gradually reached a plateau, indicating the formation of an elastic-dominant hydrogel network.

Steady-shear measurements showed that the apparent viscosity decreased with increasing shear rate, demonstrating shear-thinning behavior of the precursor solutions (Fig. 3M). The L-Leu-containing formulations exhibited higher apparent viscosity than the SM formulation, with a progressive numerical increase as the nominal L-Leu concentration increased.

During the strain sweep, G′ remained higher than G″ throughout the tested strain range for all formulations, indicating predominantly elastic behavior (Fig. 3N). At elevated strain, G′ decreased to varying degrees, reflecting nonlinear strain softening and partial disruption of the hydrogel network. The L-Leu-containing groups generally exhibited higher G′ values than the SM group.

Frequency-sweep measurements further showed that G′ remained higher than G″ over the low-to-moderate angular-frequency range, supporting the elastic-dominant behavior of the photocured hydrogel networks (Fig. 3O). The storage modulus was generally higher in the L-Leu-containing groups, particularly in the SM@L700 group.

To further assess changes in the effective crosslinking state of the hydrogel network, the mechanical, swelling, spectroscopic, and rheological findings were considered collectively. Because micropore diameter and overall porosity remained comparable among the four formulations, the increase in compressive modulus after L-Leu incorporation was unlikely to result from differences in scaffold architecture. In addition, the L-Leu-containing hydrogels generally exhibited higher G′ values during strain- and frequency-sweep measurements, while G′ remained higher than G″ throughout most or all of the tested ranges. These findings suggest that L-Leu incorporation increased the effective network constraints and apparent crosslink density of the photocured SilMA hydrogels. However, FTIR analysis showed no distinct new absorption bands, indicating that the increased network constraints were not associated with detectable formation of additional covalent crosslinks. The higher swelling ratios of the L-Leu-containing groups further suggest that the increase in effective network constraints occurred concurrently with enhanced water affinity and hydration of the hydrogel network.

### 3.3 In vitro regulatory effects of 3D-Printed SilMA@L-Leu hydrogel scaffolds on bone repair-related cellular behaviors

Given the favorable structural and physicochemical properties of the SilMA@L-Leu scaffolds, we next examined their biological effects on two key cell types involved in bone regeneration, namely bone marrow mesenchymal stem cells (BMSCs) and human umbilical vein endothelial cells (HUVECs). These in vitro models were used to assess cytocompatibility as well as the ability of the material to modulate osteogenesis- and angiogenesis-related cellular responses.

**Fig. 4:**
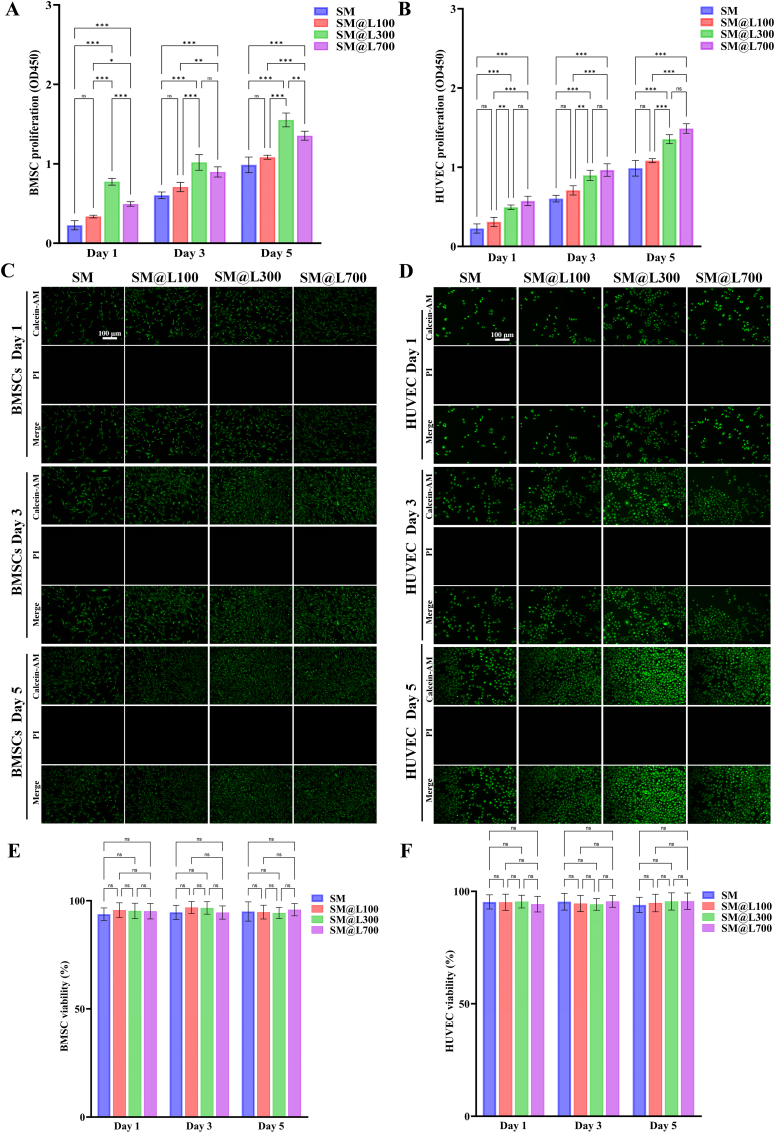
CCK-8 assays of BMSCs (A) and HUVECs (B) after 1, 3, and 5 days of culture with the corresponding scaffold extracts. C,D) Representative Live/Dead staining images of BMSCs (C) and HUVECs (D) after 1, 3, and 5 days of indirect co-culture with the scaffolds. Live cells were stained with Calcein-AM (green), whereas dead cells were stained with propidium iodide (PI; red). E,F) Quantitative analysis of BMSC viability (E) and HUVEC viability (F). SM, SilMA; SM@L100, SilMA@L-Leu-100; SM@L300, SilMA@L-Leu-300; SM@L700, SilMA@L-Leu-700. Data are presented as mean ± SD (n = 3 independent experiments per group). *p < 0.05, **p < 0.01, and ***p < 0.001; ns, not significant. Horizontal brackets indicate the groups compared. Scale bars in C and D = 100 μm. (For interpretation of the references to color in this figure legend, the reader is referred to the Web version of this article.)

CCK-8 assays and Live/Dead staining were first performed to evaluate the cytocompatibility of the SilMA@L-Leu scaffolds and their effects on cell growth (Fig. 4A–F). For both BMSCs and HUVECs, CCK-8 signals increased progressively from day 1 to day 5 across all groups, indicating that none of the formulations induced evident cytotoxic effects. Compared with pure SilMA, the L-Leu-incorporated scaffolds generally produced higher CCK-8 readings at each time point, with the difference becoming more apparent in the medium- and high-dose groups at days 3 and 5, where some comparisons reached statistical significance (P < 0.01 or P < 0.001). These data suggest that L-Leu incorporation preserved cytocompatibility while promoting cell growth in both cell types.

Live/Dead staining further supported the cytocompatibility of the scaffold system (Fig. 4C–F). In all groups, Calcein AM-positive live cells predominated, whereas PI-positive dead cells were rarely observed, indicating high cell viability across all formulations. Compared with pure SilMA, the L-Leu-loaded groups showed a broader distribution of viable cells and a higher apparent cell density. However, quantitative analysis revealed that the overall survival rate remained comparably high in all groups, with no significant intergroup differences (Fig. 4E and F). These findings indicate that L-Leu incorporation did not increase cytotoxicity and was compatible with the growth-promoting trend observed in the CCK-8 assay.

**Fig. 5:**
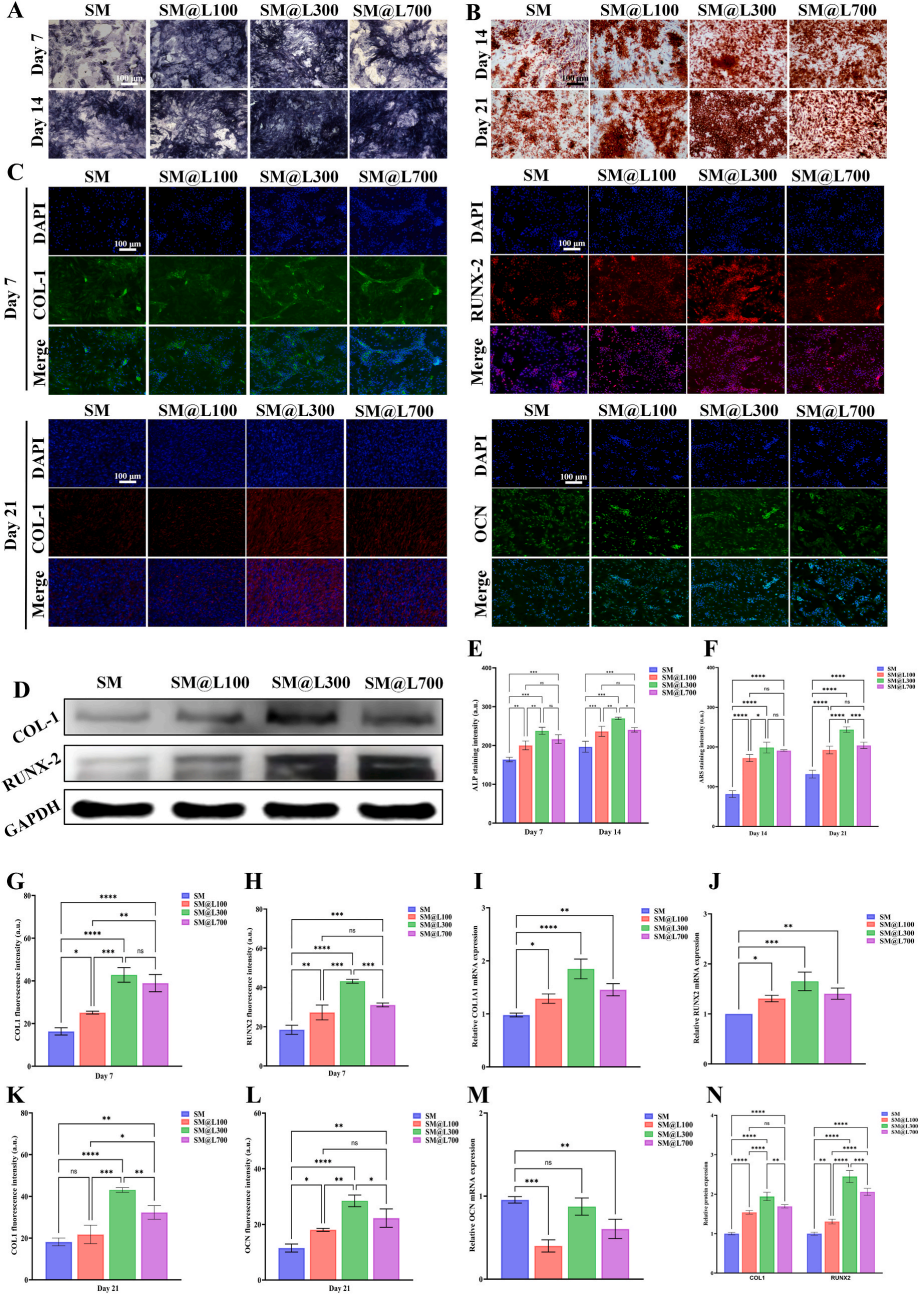
Osteogenic differentiation of BMSCs cultured with 3D-printed SilMA@L-Leu scaffolds. A) ALP staining on days 7 and 14. B) Alizarin Red S staining on days 14 and 21. C) Immunofluorescence staining of COL1, RUNX2, and OCN. D) Western blotting of COL1 and RUNX2. E,F) Quantification of ALP and mineralization. G,H) Quantification of COL1 and RUNX2 fluorescence. I,J) RT–qPCR analysis of COL1A1 and RUNX2. K,L) Quantification of COL1 and OCN fluorescence. M) RT–qPCR analysis of OCN. N) Densitometric analysis of COL1 and RUNX2. SM, SilMA; SM@L100, SM@L300, and SM@L700 indicate 100, 300, and 700 ng mL^−1^ L-Leu, respectively. Data are mean ± SD (n = 3). *p < 0.05, **p < 0.01, ***p < 0.001, ****p < 0.0001; ns, not significant. Scale bars = 100 μm. (For interpretation of the references to color in this figure legend, the reader is referred to the Web version of this article.)

After confirming good cytocompatibility, we next examined whether L-leucine (L-Leu) loading could enhance the osteogenic differentiation of BMSCs. To capture both early and late osteogenic events, ALP staining and Alizarin Red S (ARS) staining were performed at different time points (Fig. 5A,B,E,F).

ALP staining showed that L-Leu-incorporated scaffolds enhanced early osteogenic activity in BMSCs relative to pure SilMA, with the SM@L300 group displaying the strongest and most uniform staining intensity at day 14 (Fig. 5A). Quantitative analysis further confirmed that ALP activity increased from the low-to medium-dose range and reached a maximum in the SM@L300 group (Fig. 5E). Further increasing the L-Leu concentration to 700 ng mL^−1^ did not produce an additional increase, indicating that the pro-osteogenic effect of L-Leu on early-stage differentiation was most pronounced at an intermediate dose.

The late-stage mineralization results followed a similar trend. ARS staining showed that, by day 21, the SM@L300 group exhibited denser calcium nodule deposition than the other groups (Fig. 5B). Quantitative analysis further confirmed a higher level of matrix mineralization in the SM@L300 group compared with the SM and low-dose groups (Fig. 5F), indicating that an intermediate dose of L-Leu was also more favorable for late-stage osteogenic mineralization.

To further link the observed osteogenic phenotypes with underlying molecular changes, we examined the expression of representative osteogenesis-related markers. Immunofluorescence staining showed that RUNX2 and COL1 signals were enhanced in the L-Leu-loaded groups, accompanied by stronger osteogenic-marker-associated fluorescence overall, with the SM@L300 group showing the most prominent signal intensity among all formulations (Fig. 5C–G,H,K,L). These findings are consistent with the phenotypic data and suggest that an intermediate dose of L-Leu more effectively supported osteogenic progression in BMSCs.

qPCR and Western blot analyses further supported this trend at the transcriptional and protein levels (Fig. 5D–I,J,M,N). Compared with pure SilMA, L-Leu loading generally upregulated the mRNA expression of RUNX2 and COL1A1, with the strongest induction observed in the SM@L300 group. Increasing the concentration to 700 ng mL^−1^ did not result in further enhancement. Consistently, Western blotting showed elevated RUNX2 and COL1 protein levels after L-Leu incorporation, again with the most pronounced effect in the SM@L300 group, whereas the SM@L700 group offered no additional benefit. These data indicate that L-Leu promoted the osteogenic molecular program of BMSCs most effectively at an intermediate dose.

In contrast, the late osteogenic marker OCN did not show the same monotonic pattern. Within the present observation window, OCN mRNA expression did not differ significantly between the SM@L300 group and the SM group, and even declined in some dose groups (Fig. 5M), suggesting that the regulatory effects of L-Leu on osteogenic markers were not fully synchronized across differentiation stages.

**Fig. 6:**
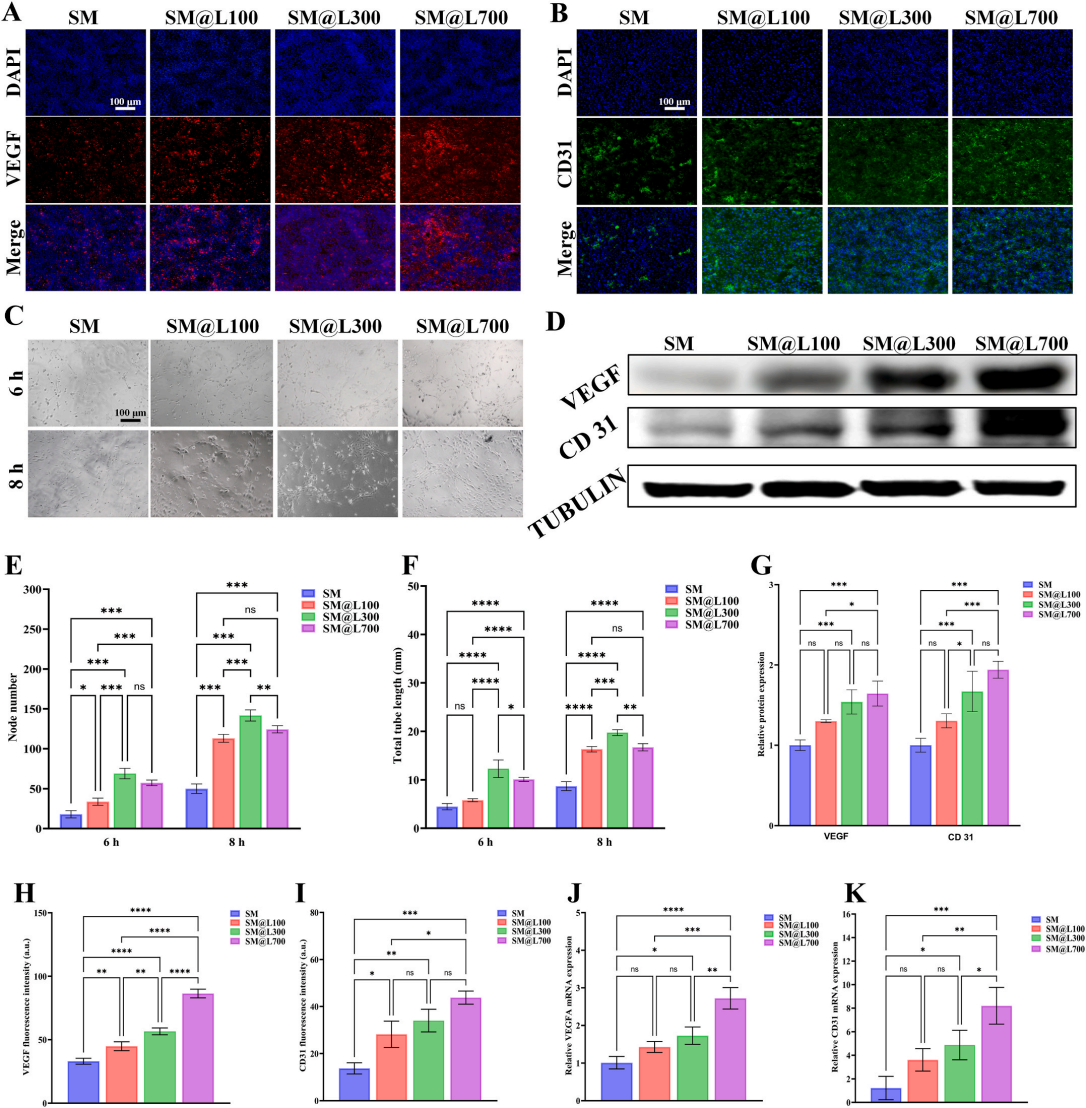
Angiogenesis-related responses of HUVECs cultured with 3D-printed SilMA@L-Leu scaffolds. A,B) Immunofluorescence staining of VEGF and CD31. C) Matrigel tube formation at 6 and 8 h. D) Western blotting of VEGF and CD31. E,F) Quantification of node number and total tube length. G) Densitometric analysis of VEGF and CD31 protein expression. H,I) Quantification of VEGF and CD31 fluorescence intensity. J,K) RT–qPCR analysis of VEGFA and CD31 expression. SM, SilMA; SM@L100, SM@L300, and SM@L700 indicate scaffolds containing 100, 300, and 700 ng mL^−1^ L-Leu, respectively. Data are mean ± SD (n = 3). *p < 0.05, **p < 0.01, ***p < 0.001, ****p < 0.0001; ns, not significant. Scale bars = 100 μm.

To evaluate the pro-angiogenic activity of SilMA@L-Leu, endothelial responses were first examined at the molecular level by immunofluorescence, Western blotting, and RT-qPCR, followed by functional assessment using a Matrigel tube formation assay (Fig. 6A–K). Relative to pure SilMA, L-Leu incorporation generally enhanced the expression of angiogenesis-related markers in HUVECs, including VEGF and CD31, with increased VEGF- and CD31-related expression compared with pure SilMA. Immunofluorescence staining showed stronger VEGF- and CD31-associated signals in the L-Leu-loaded groups, and this trend was further supported by quantitative fluorescence analysis, Western blotting, and RT-qPCR. Among the L-Leu-containing formulations, the SM@L700 group tended to exhibit stronger molecular responses for some endothelial markers, suggesting that higher L-Leu loading more robustly stimulated angiogenesis-related signaling.

We next asked whether this molecular activation translated into improved vascular morphogenesis. Matrigel tube formation showed that all L-Leu-loaded groups promoted HUVEC network assembly relative to the SM group, with more evident cell–cell connection and tubular organization (Fig. 6C). However, topological analysis revealed that the SM@L300 group formed the most integrated vascular-like networks, showing overall superior node number and total tube length at both 6 and 8 h (Fig. 6E and F). By contrast, although the SM@L700 group showed stronger molecular readouts, it did not exhibit further improvement in tube-formation topology. These findings indicate that the pro-angiogenic effects of L-Leu were not fully synchronized between molecular activation and functional vascular organization. Under the present conditions, SM@L300 provided the most favorable balance between endothelial activation and vascular-like network formation. Together with the osteogenic data, these results supported the selection of SM@L300 for subsequent in vivo studies.

**Fig. 7:**
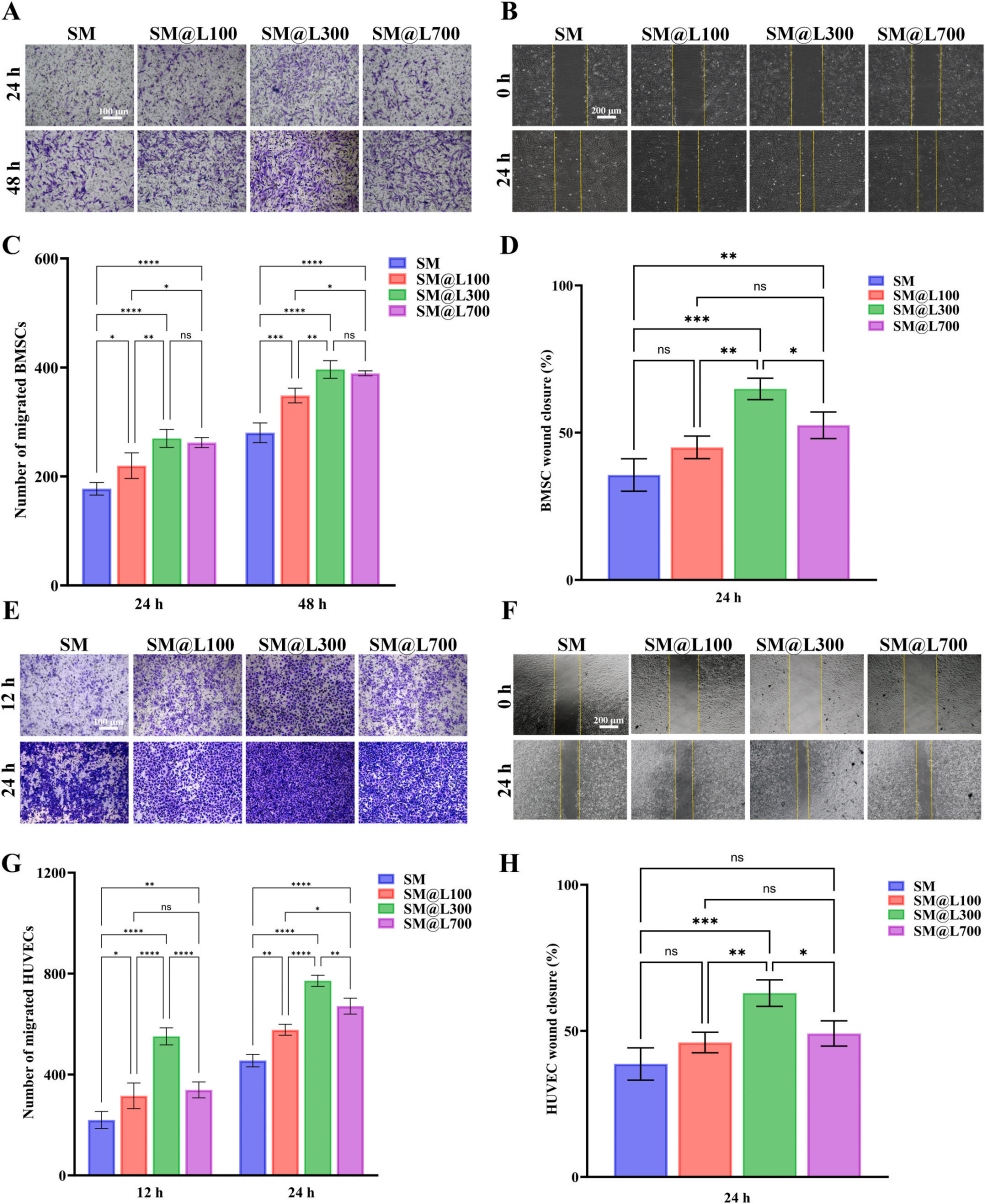
Effects of 3D-printed SilMA@L-Leu scaffolds on BMSC and HUVEC migration. A) Transwell migration of BMSCs at 24 and 48 h. B) Scratch-wound assay of BMSCs at 0 and 24 h. C,D) Quantification of migrated BMSCs and wound closure. E) Transwell migration of HUVECs at 12 and 24 h. F) Scratch-wound assay of HUVECs at 0 and 24 h. G,H) Quantification of migrated HUVECs and wound closure. SM, SilMA; SM@L100, SM@L300, and SM@L700 indicate scaffolds containing 100, 300, and 700 ng mL^−1^ L-Leu, respectively. Data are mean ± SD (n = 3). *p < 0.05, **p < 0.01, ***p < 0.001, ****p < 0.0001; ns, not significant. Scale bars = 100 μm.

After establishing that SilMA@L-Leu supported cell survival and enhanced osteogenic and angiogenic phenotypes, we next assessed its effects on cell migration, an essential event during early tissue repair (Fig. 7A–H). In BMSCs, Transwell assays showed that all L-Leu-loaded groups increased the number of migrated cells relative to pure SilMA, with the difference already apparent at 24 h and becoming more pronounced at 48 h (Fig. 7A–C). Among these groups, SM@L300 consistently produced the strongest migratory response, whereas SM@L700 did not provide further enhancement. This trend was further supported by the scratch-wound assay, in which the L-Leu-loaded groups showed greater wound closure overall and the SM@L300 group exhibited the largest closure extent at 24 h (Fig. 7B–D).

A similar pro-migratory effect was observed in HUVECs. In the Transwell assay, L-Leu incorporation increased endothelial cell migration at both 12 and 24 h, with the SM@L300 group again showing the highest number of migrated cells (Fig. 7E–G). Consistently, scratch-wound assays showed more complete wound closure in the L-Leu-loaded groups, with the most pronounced effect in the SM@L300 group at 24 h (Fig. 7F–H). Together, these results indicate that SilMA@L-Leu enhanced the migratory capacity of both osteogenic and endothelial cell populations, with SM@L300 showing the most consistent effect across assays and cell types. Taken together, the dose–response profiles were endpoint-dependent rather than uniformly favoring a single formulation. The SM@L700 group showed the greatest changes in compressive modulus, surface wettability, and selected endothelial molecular markers, whereas the SM@L300 group showed a more consistent combination of osteogenic differentiation, functional tube-network formation, and BMSC/HUVEC migration responses. Because the subsequent studies focused on combined osteogenic and angiogenic activity rather than maximization of an individual material or molecular parameter, SM@L300 was selected as a representative formulation for mechanistic and in vivo evaluation. This selection should not be interpreted as evidence that SM@L300 was globally optimal across all measured outcomes.

### 3.4 Experimental evaluation of 3D-Printed SilMA@L-Leu hydrogel scaffolds for in vivo bone defect repair

**Fig. 8:**
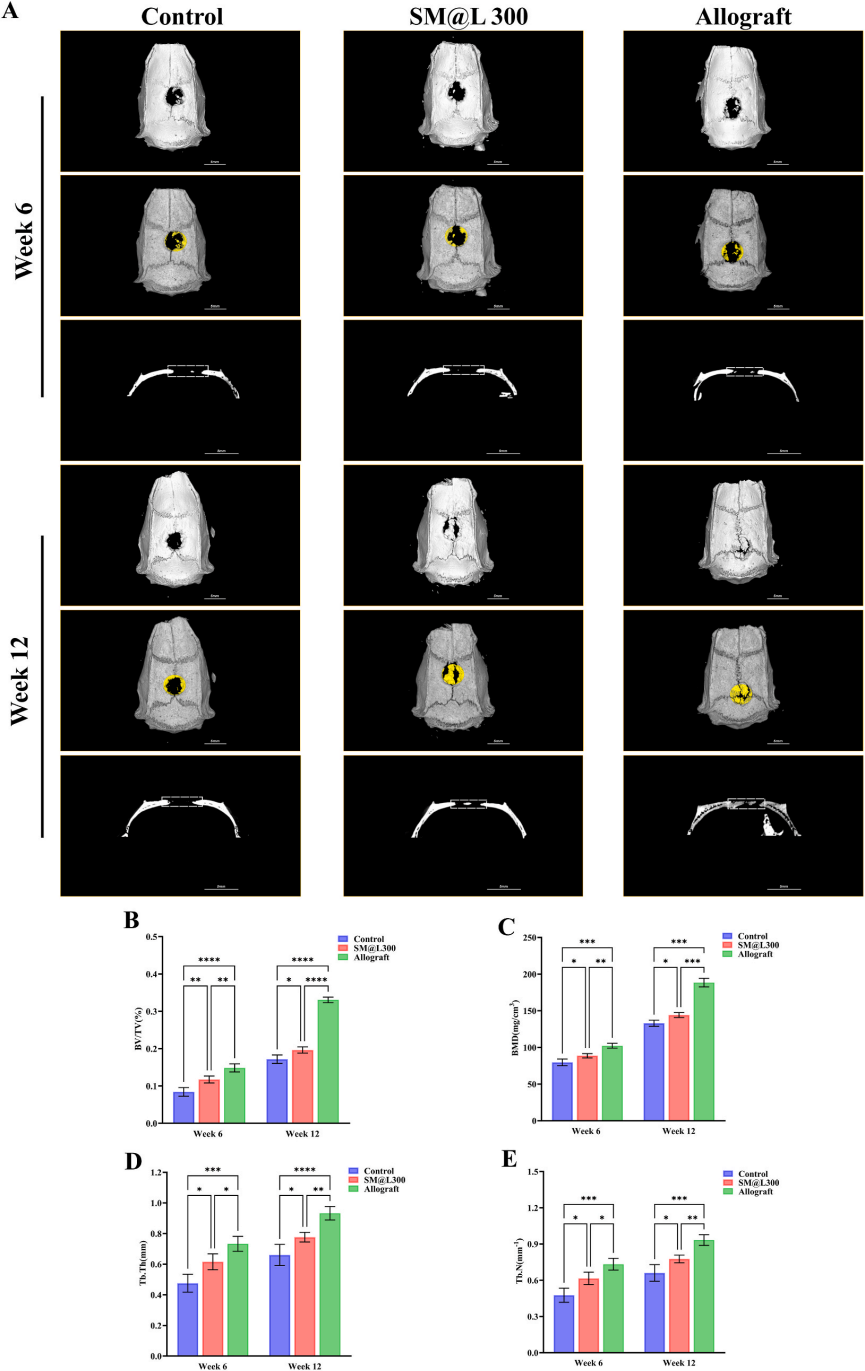
In vivo bone regeneration induced by the 3D-printed SM@L300 scaffold in rat calvarial defects. (A) Representative micro-CT reconstructions and cross-sectional images of the Control, SM@L300, and Allograft groups at 6 and 12 weeks after implantation. (B–E) Quantitative analyses of bone volume fraction (BV/TV), bone mineral density (BMD), trabecular thickness (Tb.Th), and trabecular number (Tb.N), respectively. Data are presented as mean ± SD (n = 3 animals per group at each time point). Each animal was considered an independent biological replicate. Statistical significance was assessed by two-way ANOVA followed by Tukey's multiple-comparisons test. *p < 0.05, **p < 0.01, ***p < 0.001, and ****p < 0.0001; ns, not significant. Horizontal brackets indicate the groups compared.

Based on the combined functional biological results and the selection rationale described above, the SM@L300 formulation was carried forward as a representative formulation for in vivo evaluation in a rat critical-size calvarial defect model, with the Control and allografts serving as the negative and positive controls, respectively. Micro-CT reconstruction and cross-sectional imaging showed that mineralized tissue in the Control group remained largely restricted to the defect margins at both 6 and 12 weeks (Fig. 8A). In the SM@L300 group, a greater extent of mineralized tissue was visible within the defect at 6 weeks, and more continuous mineralized bridging was observed at 12 weeks. The allograft group showed the greatest extent of mineralized defect filling at both time points.

Quantitative micro-CT analysis was interpreted according to the individual parameters shown in Fig. 8B–E. BV/TV, representing the proportion of mineralized bone volume within the region of interest, was significantly higher in the SM@L300 group than in the Control group at both 6 and 12 weeks (Fig. 8B). BMD, reflecting the mineral density of the regenerated tissue, was also higher in the SM@L300 group and increased over time (Fig. 8C). In addition, the SM@L300 group showed higher Tb.Th and Tb.N values than the Control group, indicating thicker and more numerous trabecular-like structures within the regenerated region (Fig. 8D and E). Thus, the micro-CT findings support increases in mineralized bone volume, mineral density, trabecular thickness, and trabecular number rather than a nonspecific improvement in bone repair.

**Fig. 9:**
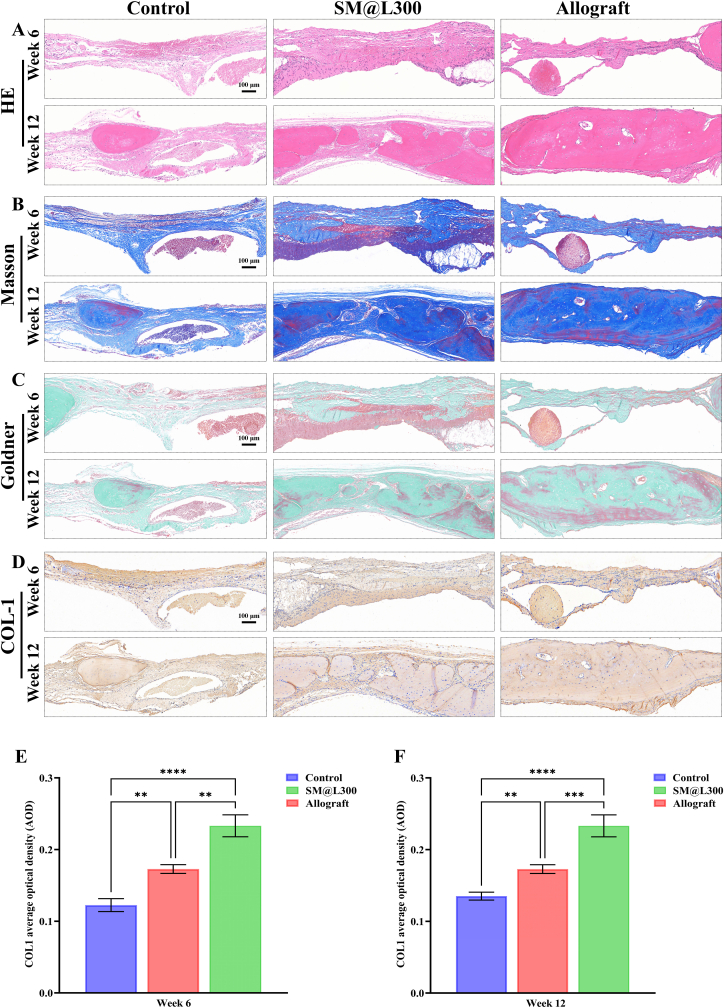
Histological and immunohistochemical evaluation of bone regeneration in rat calvarial defects. (A) Representative H&E-stained sections showing tissue morphology and defect bridging in the Control, SM@L300, and Allograft groups at 6 and 12 weeks after implantation. (B) Masson's trichrome staining showing collagen-rich matrix deposition. (C) Goldner's trichrome staining showing osteoid and mineralized matrix formation. (D) Representative immunohistochemical staining for COL1. (E,F) Quantification of the average optical density of COL1-positive staining at 6 and 12 weeks, respectively. Data are presented as mean ± SD (n = 3 animals per group at each time point). Statistical significance was determined by one-way ANOVA followed by Tukey's multiple-comparisons test. **p < 0.01, ***p < 0.001, and ****p < 0.0001. Scale bars = 100 μm.

Histological analyses provided complementary evidence of bone regeneration at 6 and 12 weeks (Fig. 9A–F). H&E staining showed that, at 6 weeks, the defects in the Control group were predominantly filled with loosely organized fibrous connective tissue, with limited osteoid-like tissue near the defect margins. In comparison, the SM@L300 group exhibited more extensive tissue ingrowth and bone-like trabecular structures extending from the defect margins toward the defect center. The Allograft group also showed substantial tissue filling. At 12 weeks, more continuous bone-like tissue bridging was observed in the SM@L300 group, whereas a considerable unrepaired region remained in the Control group. The Allograft group showed the most extensive and continuous bone-like tissue filling within the defect area (Fig. 9A).

Masson's trichrome staining revealed denser and more continuous collagen-rich matrix in the SM@L300 group than in the Control group at both time points. The collagen-rich regions became broader and more organized from 6 to 12 weeks. The Allograft group exhibited the most extensive collagen-rich matrix distribution, particularly at 12 weeks (Fig. 9B). Consistently, Goldner's trichrome staining showed more extensive mineralized matrix formation in the SM@L300 group than in the Control group, whereas the Allograft group exhibited the greatest extent of mineralized tissue and defect filling at both time points (Fig. 9C).

COL1 immunohistochemical staining showed stronger and more broadly distributed positive signals in the SM@L300 and Allograft groups than in the Control group (Fig. 9D). Quantitative analysis of the average optical density (AOD) confirmed that COL1 expression was significantly higher in the SM@L300 group than in the Control group at both 6 and 12 weeks (p < 0.01). The Allograft group showed the highest COL1 AOD, with significantly higher values than the Control group at both time points (p < 0.0001) and significantly higher values than the SM@L300 group at 6 weeks (p < 0.01) and 12 weeks (p < 0.001) (Fig. 9E and F). These findings indicate that SM@L300 enhanced type I collagen deposition during calvarial defect repair, although its effect remained lower than that of the Allograft group.

**Fig. 10:**
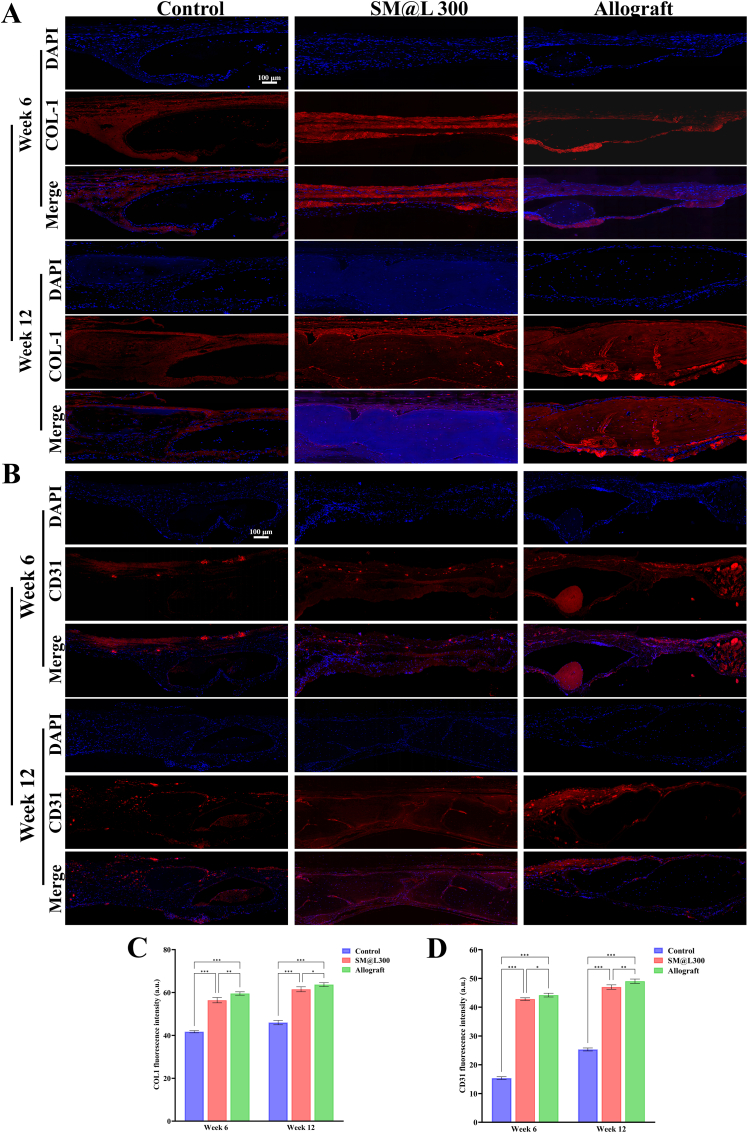
Immunofluorescence evaluation of COL1 and CD31 in rat calvarial defects. (A,B) Representative immunofluorescence images of COL1 (A) and CD31 (B) in the Control, SM@L300, and Allograft groups at 6 and 12 weeks after implantation. COL1 and CD31 are shown in red, and nuclei are counterstained with DAPI (blue). C,D) Quantitative analysis of the mean fluorescence intensities of COL1 (C) and CD31 (D). Data are presented as mean ± SD (n = 3 animals per group at each time point). Statistical significance was determined by one-way ANOVA followed by Tukey's multiple-comparisons test. *p < 0.05, **p < 0.01, and ***p < 0.001. Scale bars = 100 μm. (For interpretation of the references to color in this figure legend, the reader is referred to the Web version of this article.)

Immunofluorescence analysis showed that COL1 and CD31 signals followed the same pattern at 6 and 12 weeks: Control < SM@L300 < Allograft (Fig. 10A–D). Both markers were significantly higher in the SM@L300 and Allograft groups than in the Control group at both time points (all p < 0.001). The Allograft group also showed higher COL1 intensity than SM@L300 at 6 and 12 weeks (p < 0.01 and p < 0.05, respectively) and higher CD31 intensity (p < 0.05 and p < 0.01, respectively). These findings indicate that SM@L300 enhanced collagen deposition and endothelial-associated signals, although the effects remained lower than those of the Allograft group.

**Fig. 11:**
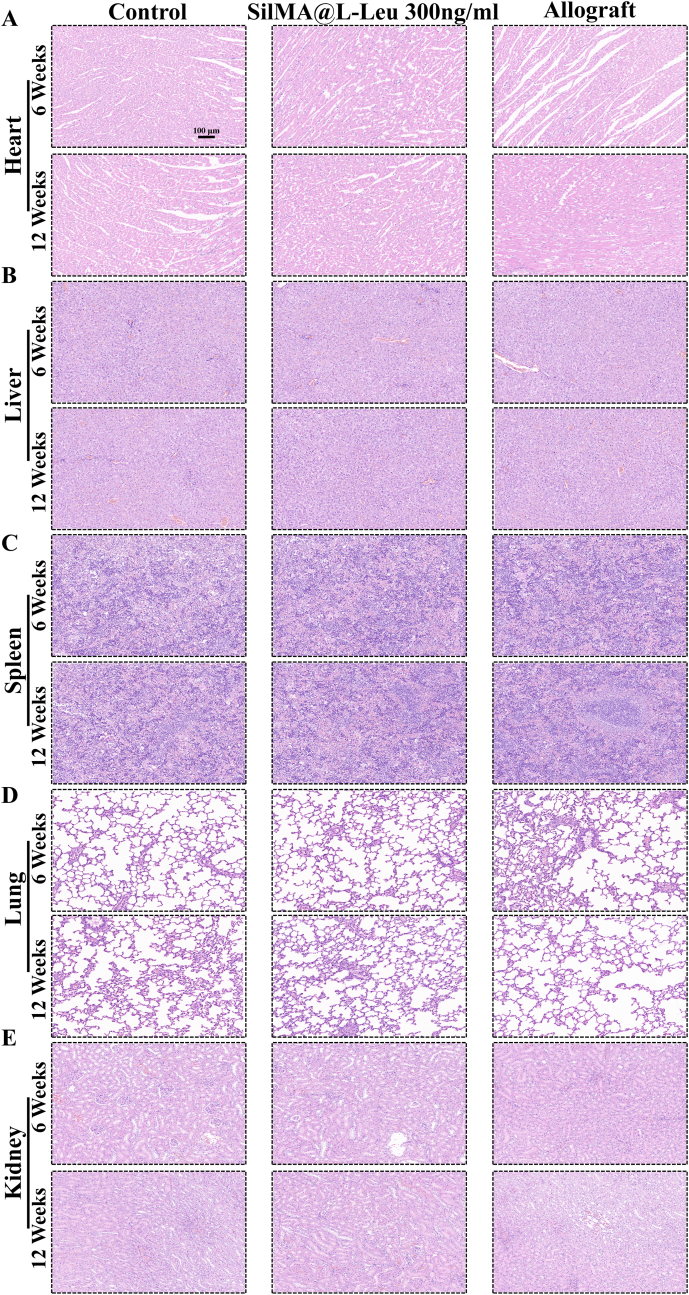
Preliminary histological safety assessment of major organs after scaffold implantation. Representative H&E-stained sections of the heart, liver, spleen, lung, and kidney from the Control, SM@L300, and Allograft groups at 6 and 12 weeks after implantation. No overt tissue necrosis, marked inflammatory infiltration, or major structural abnormalities were observed in the representative sections. Images were obtained from three animals per group at each time point, with each animal considered an independent biological replicate. No inferential statistical analysis was performed because the assessment was qualitative. Scale bars = 100 μm.

A preliminary histological safety assessment was performed by H&E staining of the heart, liver, spleen, lung, and kidney at 6 and 12 weeks postoperatively (Fig. 11A–E). In the representative sections examined, the general tissue architecture of these organs appeared preserved across the Control, SM@L300, and Allograft groups. No overt necrosis, marked inflammatory infiltration, or major structural disorganization was observed, and no obvious qualitative intergroup differences were identified at either time point. These observations provide preliminary evidence that implantation of SilMA@L-Leu was not associated with overt histopathological abnormalities in the examined organs during the present observation period. However, they do not establish comprehensive systemic biosafety or exclude subclinical and functional toxicity.

### 3.5 Involvement of PLOD2-Associated collagen matrix remodeling in the pro-osteogenic effect of 3D-Printed SilMA@L-Leu hydrogel scaffolds

**Fig. 12:**
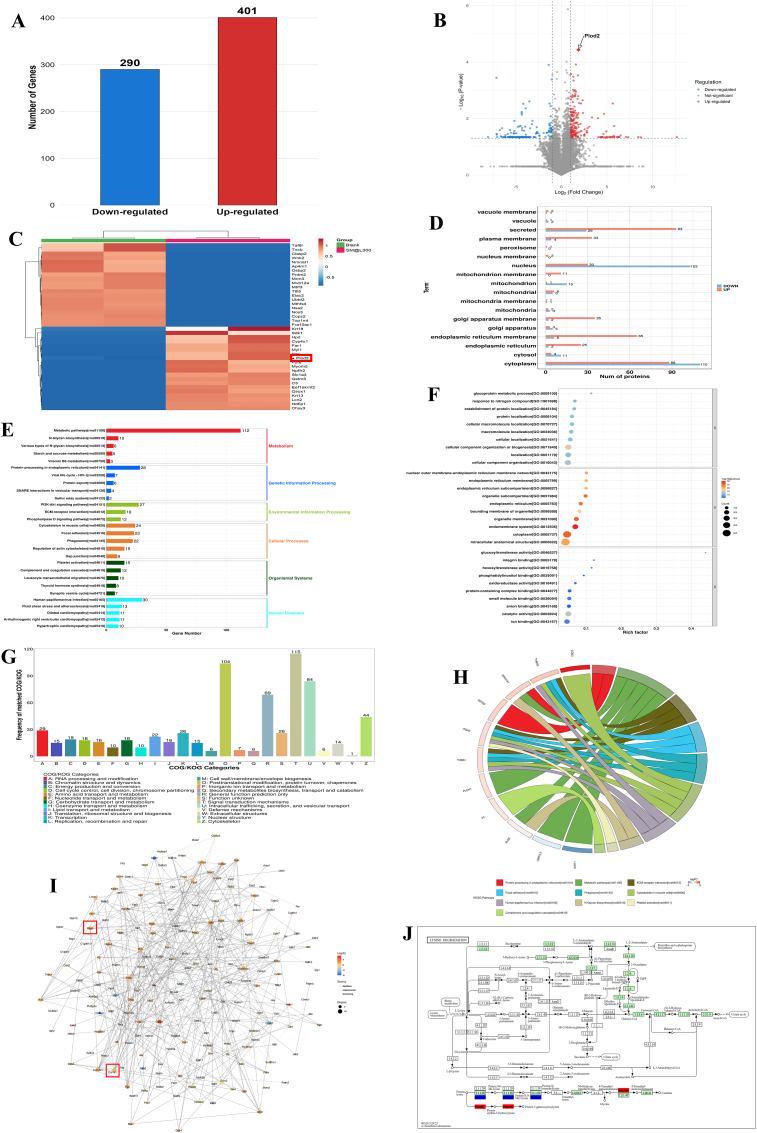
Comparative proteomic profiling of Control and SM@L300-treated BMSCs. (A) Numbers of upregulated and downregulated proteins in the SM@L300 group relative to the Control group. (B) Volcano plot of differentially expressed proteins, with PLOD2 highlighted. (C) Hierarchical clustering heatmap of representative differentially expressed proteins. (D) Subcellular localization analysis of the differentially expressed proteins. (E) KEGG pathway classification. (F) Gene Ontology enrichment analysis. (G) COG/KOG functional classification. (H) Pathway–protein association chord diagram. (I) Protein–protein interaction network, with PLOD2 and COL1A1 highlighted. (J) KEGG pathway map showing PLOD2 and COLGALT1 within the lysine-modification-related branch associated with collagen hydroxylysine formation and glycosylation.

Given that SM@L300 enhanced osteogenic phenotypes in vitro and promoted calvarial defect repair in vivo, we next explored the underlying molecular basis by comparative proteomic analysis of BMSCs in the Control and SM@L300 groups (Fig. 12). A total of 691 differentially expressed proteins were identified in the SM@L300 group relative to the Control group, including 401 upregulated and 290 downregulated proteins (Fig. 12A). Volcano-plot analysis showed a clear separation of differentially expressed proteins and highlighted PLOD2 as an upregulated candidate protein (Fig. 12B). Hierarchical clustering further demonstrated distinct protein-expression patterns between the two groups, with PLOD2 included among the representative upregulated proteins in the SM@L300 group (Fig. 12C).

Subcellular localization analysis indicated that the differentially expressed proteins were distributed across the cytoplasm, nucleus, mitochondria, plasma membrane, endoplasmic reticulum, extracellular region, and other cellular compartments (Fig. 12D). KEGG classification, GO enrichment, and COG/KOG functional annotation showed that these proteins were involved in metabolic processes, protein processing, intracellular transport, signal transduction, cytoskeletal regulation, and extracellular matrix-related functions (Fig. 12E–G). Pathway–protein association analysis further demonstrated that representative differentially expressed proteins participated in multiple interconnected biological pathways (Fig. 12H). Protein–protein interaction network analysis identified associations among proteins involved in extracellular matrix organization and collagen processing, with PLOD2 and COL1A1 highlighted as candidate proteins of interest (Fig. 12I). KEGG pathway mapping further placed PLOD2 and COLGALT1 within a lysine-modification-related branch associated with collagen hydroxylysine formation and subsequent glycosylation (Fig. 12J). Collectively, these findings supported the selection of PLOD2 for subsequent loss-of-function validation.

**Fig. 13:**
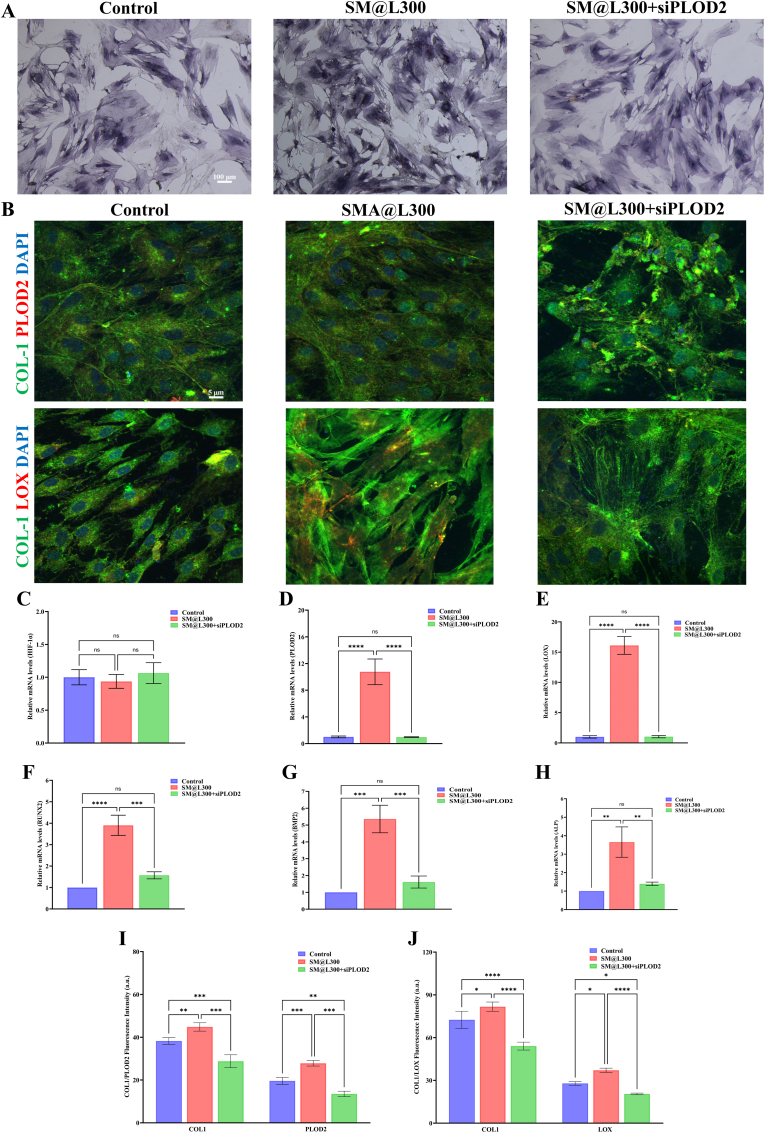
Involvement of PLOD2-associated extracellular matrix remodeling in the pro-osteogenic effect of SM@L300. (A) ALP staining of BMSCs in the Control, SM@L300, and SM@L300 + siPLOD2 groups. (B) Immunofluorescence staining of COL1, PLOD2, and LOX. (C–H) RT–qPCR analysis of HIF-1α, PLOD2, LOX, RUNX2, BMP2, and ALP expression, respectively. (I) Quantification of COL1 and PLOD2 fluorescence intensity. (J) Quantification of COL1 and LOX fluorescence intensity. Data are presented as mean ± SD (n = 3 independent experiments per group). *p < 0.05, **p < 0.01, ***p < 0.001, and ****p < 0.0001; ns, not significant. Horizontal brackets indicate the groups compared. Scale bars = 100 μm.

To determine whether the pro-osteogenic effect of SilMA@L-Leu was linked to PLOD2-associated ECM remodeling, we compared Control, SM@L300, and SM@L300 + siPLOD2 groups (Fig. 13). ALP staining showed that SilMA@L-Leu markedly enhanced early osteogenic activity, whereas this effect was substantially attenuated after PLOD2 knockdown (Fig. 13A). Consistently, immunofluorescence analysis showed increased COL1 deposition together with elevated PLOD2 and LOX signals in the SM@L300 group, while siPLOD2 reduced PLOD2 expression and was accompanied by weakened COL1 and LOX signals (Fig. 13B–I,J). At the transcriptional level, qRT-PCR further confirmed effective knockdown of PLOD2 and concurrent reduction of LOX expression (Fig. 13D and E). Moreover, the osteogenesis-related genes RUNX2, BMP2, and ALP were upregulated by SilMA@L-Leu treatment but were all reduced to varying degrees after PLOD2 silencing, with expression levels tending back toward those of the control group (Fig. 13F–H). In contrast, HIF-1α did not differ significantly among groups (Fig. 13C). Together, these findings suggest that SilMA@L-Leu-induced osteogenic differentiation is associated with PLOD2-related collagen matrix remodeling. The accompanying reductions in LOX expression and COL1 deposition after PLOD2 silencing indicate broader changes in collagen maturation and extracellular matrix organization. However, these concomitant changes do not establish LOX as a direct downstream target or functional mediator of PLOD2. In the present study, LOX was evaluated as an ECM-remodeling-associated collagen maturation and crosslinking marker. Although PLOD2 silencing attenuated the SilMA@L-Leu-induced osteogenic and matrix-remodeling responses, these effects were not demonstrated to be completely abolished. Therefore, the present findings support a contributory role for PLOD2-associated collagen matrix remodeling but do not establish complete PLOD2 dependency.

## 4 Discussion

Effective repair of complex bone defects requires scaffolds that provide structural support while regulating cell behavior, extracellular matrix remodeling, and vascularization [18]. In this study, a photocurable, 3D-printed SilMA scaffold incorporating L-Leu maintained printability and structural integrity while improving compressive properties, hydration, and surface wettability. These physicochemical changes were accompanied by enhanced osteogenic and angiogenesis-related responses and improved bone repair in vivo. Because scaffold geometry remained comparable across groups, the observed biological effects were more likely associated with L-Leu-mediated compositional and interfacial optimization than with differences in scaffold architecture [19].

L-Leu incorporation did not compromise scaffold printability or structural fidelity, as all formulations maintained well-defined porous architectures, preserved macroscopic geometry, and aligned internal channels [20]. Micropore size and porosity were also comparable among groups, indicating that L-Leu did not measurably alter the overall scaffold architecture [21]. This structural consistency is important because it reduces geometry-related confounding and suggests that the observed biological effects were primarily associated with compositional and interfacial changes introduced by L-Leu [22]. Previous studies have similarly shown that scaffold functionalization can enhance cell–material interactions and regenerative performance without substantially changing pore architecture or print fidelity [23].

Against the background of a largely preserved porous architecture, L-Leu incorporation altered several key physicochemical properties of the SilMA scaffolds. Compared with pristine SilMA, the L-Leu-containing scaffolds exhibited higher compressive moduli and lower water contact angles, indicating improved mechanical support and surface wettability. The L-Leu-containing groups also showed numerically higher swelling ratios at later time points, suggesting a possible enhancement in hydration capacity. In bone tissue engineering, mechanical stability, water uptake, and surface wettability collectively influence structural support at the defect site, fluid infiltration, nutrient transport, protein adsorption, and early cell–material interactions [24]. Therefore, the effects of L-Leu may be better understood as a coordinated optimization of overall scaffold functionality rather than an isolated change in a single material parameter. This integrated property profile may enable the scaffold to maintain structural stability while providing a more hydrophilic interface that is favorable for cell adhesion and mass transport [25].

Although the L-Leu-containing scaffolds showed slightly lower mass retention than pristine SilMA at day 63, all formulations exhibited slow and broadly comparable mass-loss profiles and retained a substantial proportion of their initial mass throughout the observation period. These findings indicate that L-Leu incorporation did not cause rapid scaffold disintegration or markedly compromise long-term structural stability [26]. The L-Leu release profiles showed an initial rapid-release phase during the first 1–3 days, followed by a slower release stage extending to day 28 (Fig. 3H). The initial release may be attributed to L-Leu located near the scaffold surface or weakly confined within the outer regions of the polymer network, whereas the subsequent slower phase may reflect diffusion through the photocrosslinked SilMA network and reversible noncovalent interactions between L-Leu and SilMA [27]. Therefore, although L-Leu was physically incorporated rather than covalently conjugated, it was not completely released immediately after immersion (Fig. 3H).

The three L-Leu-containing formulations exhibited comparable encapsulation efficiencies of approximately 81%–84% (Fig. 3I), indicating that the differences in their biological performance were unlikely to result primarily from unequal incorporation efficiency. Although SM@L700 showed relatively greater L-Leu retention at later time points, it did not consistently produce superior osteogenic or functional angiogenic responses. In contrast, SM@L300 showed a more favorable balance among L-Leu availability, continued retention, osteogenic differentiation, functional endothelial tube formation, and BMSC/HUVEC migration. Thus, SM@L300 was selected as the most suitable formulation for subsequent mechanistic and in vivo evaluation among the tested conditions. This selection was based on the balance between release characteristics and functional biological performance rather than on the highest encapsulation efficiency or the slowest release profile alone. In addition, the pH of the immersion medium remained close to neutral during prolonged incubation (Fig. 3J), suggesting that L-Leu release did not markedly disturb the acid–base balance of the surrounding environment.

Importantly, the release experiment was conducted for 28 days, whereas the in vivo regenerative outcomes were evaluated at 6 and 12 weeks. Therefore, the present results do not demonstrate continuous L-Leu release throughout the entire in vivo observation period, and the long-term regenerative effects should not be attributed solely to persistent L-Leu exposure. Instead, the early and gradual local availability of L-Leu may have initiated sustained changes in cell behavior and extracellular matrix remodeling, including PLOD2-associated collagen remodeling, while the remaining photocrosslinked SilMA scaffold continued to provide structural and interfacial support [28]. Nevertheless, direct in vivo quantification of residual L-Leu and longer-term release studies will be required to verify this interpretation.

FTIR analysis revealed the characteristic chemical-bond vibrations of the SilMA matrix, including the O–H/N–H stretching band and the amide I and amide II bands, which were mainly associated with C=O stretching and N–H bending/C–N stretching, respectively. After L-Leu incorporation, the principal peak positions and overall spectral profiles remained largely unchanged, and no distinct new absorption bands were observed. These findings indicate that L-Leu loading did not induce detectable formation of additional covalent bonds or disrupt the primary photocrosslinked framework of SilMA [29]. Nevertheless, subtle variations in peak shape and relative intensity within the O–H/N–H-associated region suggest that the amino and carboxyl groups of L-Leu may interact with the amide, hydroxyl, and carbonyl groups of the SilMA chains through hydrogen bonding [30]. These reversible hydrogen-bonding interactions may function as supplementary physical crosslinking points within the covalently photocrosslinked SilMA network [31]. By enhancing intermolecular association and partially restricting polymer-chain mobility, these physical interactions may increase the effective network constraints and apparent crosslink density of the hydrogel [27]. This interpretation is consistent with the higher compressive modulus and the generally elevated G′ values observed in the L-Leu-containing groups [32]. Because micropore diameter and overall porosity remained comparable among formulations, the mechanical differences were unlikely to result primarily from changes in scaffold architecture [33]. Rheological measurements further supported changes in the effective network structure after L-Leu incorporation [34]. Following 405-nm irradiation, both G′ and G″ increased rapidly, confirming rapid photocrosslinking of all formulations, while the subsequent predominance of G′ indicated the formation of elastic-dominant hydrogel networks [35]. The higher apparent viscosity of the L-Leu-containing precursor solutions may be attributed, at least in part, to hydrogen-bond-mediated intermolecular association between L-Leu and SilMA. As the shear rate increased, partial dissociation of these reversible hydrogen bonds, together with polymer-chain rearrangement, may have reduced flow resistance and resulted in shear-thinning behavior [36]. During strain-sweep testing, G′ remained higher than G″ throughout the tested range, while the decrease in G′ at elevated strain indicated nonlinear strain softening and partial network disruption [36]. This behavior may reflect the progressive dissociation of reversible physical interactions, including hydrogen bonds, under large deformation. Frequency-sweep measurements similarly showed elastic-dominant behavior over the low-to-moderate angular-frequency range [37]. Taken together with the compression results, these findings suggest that the covalent network formed by SilMA photopolymerization provided the primary structural framework, whereas L-Leu-mediated hydrogen bonds acted as supplementary physical crosslinks that increased effective network constraints and contributed to the altered viscoelastic response [31]. Notably, the proposed increase in effective network constraints does not necessarily conflict with the higher swelling ratios observed in the L-Leu-containing groups [33]. In addition to forming intermolecular hydrogen bonds with SilMA, the polar amino and carboxyl groups of L-Leu may interact with water molecules and enhance water retention within the scaffold. This increased affinity for water is consistent with the observed tendency toward higher swelling and the marked reduction in water contact angle [38]. Therefore, the swelling behavior likely reflects a balance between increased physical network constraints and enhanced thermodynamic affinity for water. Overall, the results suggest that L-Leu incorporation did not measurably increase the chemical crosslink density through the formation of new covalent bonds, but may have increased the effective or apparent crosslink density through reversible hydrogen-bond-mediated physical interactions [39].

The materials-level optimization translated into clear cellular benefits. SilMA@L-Leu maintained favorable cytocompatibility in both BMSCs and HUVECs, as indicated by the high proportion of viable cells and the absence of significant intergroup differences in overall survival [40]. Meanwhile, CCK-8 assays showed consistently higher activity readouts in the L-Leu-loaded groups for both cell types, suggesting that the primary effect was not improved survival per se, but enhanced cellular activity and population expansion. Previous studies likewise indicate that optimization of the scaffold microenvironment is often first reflected in enhanced adhesion, metabolic activity, and proliferative capacity rather than simply reduced cell death [41]. Notably, the dose-response patterns differed between BMSCs and HUVECs: BMSCs responded most favorably at an intermediate dose, whereas HUVECs showed a clearer dose-dependent increase at selected time points. This cell-type-specific response suggests that L-Leu may differentially regulate repair-relevant cell populations through modulation of the cell–material interface. Similar cell-specific effects have been widely reported in scaffolds designed to integrate osteogenic and angiogenic functions, as different cell types do not respond uniformly to material composition, molecular cues, or interfacial properties [42]. Taken together, these findings suggest that L-Leu incorporation preserves cytocompatibility while improving the interfacial and local regenerative microenvironment, thereby supporting parallel increases in the activity of BMSCs and HUVECs. Because the two cell populations were evaluated separately, these results do not establish direct functional coupling between osteogenic and endothelial cells [43].

SilMA@L-Leu enhanced BMSC osteogenic differentiation, as supported by early osteogenic activity, matrix mineralization, and osteogenesis-related marker expression [44]. However, the dose–response relationship varied among endpoints. Whereas SM@L700 produced stronger changes in certain physicochemical properties and endothelial molecular markers, SM@L300 showed a more consistent combination of osteogenic differentiation, functional tube formation, and BMSC/HUVEC migration [45]. SM@L300 was therefore selected as a representative formulation on the basis of balanced functional performance rather than superiority in every measured parameter [46]. Because no formal multi-criteria optimization or direct in vivo comparison between SM@L300 and SM@L700 was performed, the current findings do not establish SM@L300 as universally optimal [47].

SilMA@L-Leu also enhanced Transwell migration and scratch-wound closure in both BMSCs and HUVECs [48]. Cell recruitment is important during early bone healing because infiltration of osteogenic and endothelial cells supports subsequent matrix deposition, vascularization, and tissue reconstruction [49]. Thus, the pro-migratory effects observed here suggest that the scaffold may influence not only differentiation-related responses but also early cellular recruitment [50]. Nevertheless, because these assays were performed in separate cell systems, they do not demonstrate direct communication between BMSCs and HUVECs [51].

The in vitro benefits of SilMA@L-Leu were further supported by the in vivo findings. Increased BV/TV and BMD indicated greater mineralized bone volume and density, whereas higher Tb.Th and Tb.N reflected thicker and more numerous trabecular-like structures. Together with the reconstructed images, these results support improved mineralized tissue formation and defect bridging rather than a nonspecific improvement in bone repair [52].

Histological analyses provided complementary evidence of bone regeneration. H&E staining showed greater tissue ingrowth and more continuous defect bridging in the SM@L300 group than in the Control group, whereas Masson's and Goldner's trichrome staining supported enhanced collagen-rich matrix deposition and progressive mineralized matrix formation [53]. The Allograft group generally exhibited the most extensive tissue filling, collagen matrix organization, and mineralized tissue formation, consistent with its role as the positive control。

Unlike the descriptive assessments of H&E, Masson's trichrome, and Goldner's trichrome staining, COL1 immunohistochemical staining was quantitatively evaluated. COL1 AOD was significantly higher in the SM@L300 group than in the Control group at both 6 and 12 weeks, supporting enhanced type I collagen deposition after scaffold implantation. However, the Allograft group showed the highest COL1 AOD at both time points, with values significantly exceeding those of the SM@L300 group. These findings indicate that SM@L300 promoted collagen matrix formation but did not exceed the collagen-regenerative performance of the Allograft group. Because corresponding morphometric quantification was not performed for H&E, Masson's trichrome, or Goldner's trichrome staining, conclusions based on these staining methods should remain descriptive [54].

The immunofluorescence findings in Fig. 10 further supported the effects of SM@L300 on collagen matrix formation and vascularization-related responses. Quantitative analysis demonstrated that COL1 fluorescence intensity was significantly higher in the SM@L300 group than in the Control group at both 6 and 12 weeks. This result was consistent with the COL1 immunohistochemical findings and supports the capacity of SM@L300 to promote type I collagen synthesis, deposition, and matrix remodeling within the defect region [55]. However, the Allograft group exhibited the highest COL1 fluorescence intensity at both time points, indicating that although SM@L300 enhanced collagen matrix formation, its effect remained lower than that achieved with allogeneic bone grafting. Similarly, CD31 fluorescence intensity was significantly increased in the SM@L300 group relative to the Control group at both postoperative time points, suggesting enhanced endothelial-associated activity and vascularization-related responses following scaffold implantation [56]. These in vivo observations were consistent with the in vitro findings of enhanced HUVEC migration, angiogenesis-related marker expression, and tubular network formation, collectively supporting the pro-angiogenic potential of SilMA@L-Leu [51]. Nevertheless, the Allograft group showed significantly higher CD31 fluorescence intensity than the SM@L300 group, indicating that the vascularization-related response induced by SM@L300 did not reach the level observed after allograft implantation.

H&E staining of the heart, liver, spleen, lung, and kidney showed no overt histopathological abnormalities in the representative sections examined at 6 or 12 weeks. These findings provide preliminary morphological evidence of local treatment compatibility at the major-organ level within the tested dose range and observation period. However, H&E staining alone cannot establish the absence of systemic toxicity or comprehensive in vivo biosafety. Hematological parameters, serum biochemical indicators of organ function, systemic inflammatory and immune responses, material biodistribution, and longer-term adverse effects were not evaluated in the present study [57].

Mechanistically, comparative proteomic profiling revealed extensive protein-expression remodeling in SM@L300-treated BMSCs. A total of 691 differentially expressed proteins were identified relative to the Control group, including 401 upregulated and 290 downregulated proteins. Functional classification and enrichment analyses indicated that these proteins were broadly involved in metabolic regulation, protein processing and transport, signal transduction, cytoskeletal organization, and extracellular matrix-associated processes. These findings suggest that SM@L300 does not act through a single isolated pathway but instead induces coordinated changes in cellular metabolism, protein homeostasis, cell–matrix interactions, and matrix organization [58]. Notably, PLOD2 was highlighted as an upregulated candidate in both the volcano plot and hierarchical clustering analysis. Protein–protein interaction analysis further connected PLOD2 with extracellular matrix- and collagen-associated proteins, including COL1A1. In addition, pathway mapping placed PLOD2 and COLGALT1 within a lysine-modification-related branch associated with collagen hydroxylysine formation and glycosylation. Because these post-translational modifications contribute to collagen maturation and extracellular matrix organization, the proteomic findings provided a rationale for selecting PLOD2 for subsequent loss-of-function validation. However, the proteomic analysis identifies associations rather than direct causal relationships, and the functional contribution of PLOD2 therefore required experimental validation [59]. This interpretation is also consistent with previous studies showing that interfacial optimization can reshape ECM organization and cell adhesion states, and then propagate through focal adhesion remodeling and downstream signaling into divergent osteogenic outcomes [60].

PLOD2 loss-of-function assessment further supported the involvement of collagen matrix remodeling in the osteogenic response induced by SilMA@L-Leu [28]. PLOD2 silencing attenuated ALP activity, osteogenesis-related gene expression, and COL1 deposition, indicating that PLOD2 contributes to the observed osteogenic effect but is not necessarily an indispensable or exclusive mediator [61]. The accompanying reduction in LOX expression further suggested broader changes in the collagen-processing and matrix-maturation environment [62].

PLOD2 and LOX participate in distinct but complementary stages of collagen maturation [63]. PLOD2 regulates the hydroxylation of collagen lysine residues during intracellular collagen processing, thereby influencing the formation of hydroxylysine-derived crosslinks [64]. In contrast, LOX primarily acts after collagen secretion by initiating covalent crosslink formation within the extracellular matrix [63]. In the present study, LOX was therefore evaluated as a collagen-maturation-associated marker rather than as a predefined downstream effector of PLOD2 [62]. Because LOX was not independently silenced, pharmacologically inhibited, or evaluated in a rescue experiment, the concurrent reduction in LOX expression after PLOD2 knockdown does not establish direct regulation of LOX by PLOD2 or demonstrate that LOX functionally mediates the osteogenic effect of SilMA@L-Leu [65].

Notably, HIF-1α expression did not differ significantly among groups. Therefore, although PLOD2-associated collagen remodeling was involved in the observed osteogenic response, the present results do not demonstrate detectable activation of the HIF-1α/PLOD2 axis under the tested experimental conditions.

Several limitations should be acknowledged. Although the calvarial defect model is suitable for evaluating osteogenesis and vascularization, it does not fully recapitulate the mechanical demands of load-bearing defects [62]. The performance of this scaffold in load-bearing or segmental bone defect models therefore requires further investigation. In addition, although PLOD2 emerged as a key molecular node, the upstream mechanism by which L-Leu regulates this axis remains unclear, particularly with respect to amino acid sensing or mechano-metabolic coupling [66]. Furthermore, although PLOD2 silencing attenuated the osteogenic and matrix-remodeling responses, complete PLOD2 dependency was not established because rescue experiments, independent pharmacological interventions, and in vivo PLOD2-specific loss-of-function analyses were not performed [67]. LOX was evaluated only as a collagen-maturation-associated marker; therefore, its causal role in SilMA@L-Leu-induced matrix remodeling and osteogenic differentiation requires independent functional validation [68]. This study also used a cell-free scaffold system together with relatively simplified in vitro models [65]. Moreover, BMSCs and HUVECs were evaluated in separate experimental systems, without direct or indirect co-culture, cell-specific pathway perturbation, or rescue experiments [28]. Consequently, the present findings demonstrate parallel osteogenic and angiogenic responses to SilMA@L-Leu but do not establish causal communication or functional coupling between osteoblast-lineage and endothelial cells [69]. Future studies using BMSC–endothelial co-culture systems and cell-specific intervention will be required to determine whether the scaffold promotes genuine osteovascular coupling. While this design helped isolate the intrinsic contribution of the material, it provided limited insight into immune cell participation, long-term remodeling, and refined vascular quantification [70]. Furthermore, the safety assessment was limited to qualitative H&E examination of selected major organs at 6 and 12 weeks [64]. Hematological indices, serum biochemical markers of hepatic and renal function, systemic inflammatory and immune responses, quantitative histopathological scoring, material biodistribution, and longer-term toxicity were not evaluated. Therefore, the present findings should be regarded as preliminary histological safety evidence rather than a comprehensive demonstration of systemic biosafety. Given the dynamic coupling among osteogenesis, vascularization, and the immune microenvironment during bone repair, future studies incorporating macrophage responses, longer-term in vivo evaluation, and more systematic vascular analyses would further strengthen the mechanistic depth and translational relevance of these findings [71].

Collectively, this study presents a scaffold-design strategy that integrates photocrosslinkable SilMA with a small-molecule bioactive cue to coordinate structural performance and matrix-associated biological regulation [55]. The attenuation of osteogenic responses after PLOD2 silencing suggests that PLOD2-associated collagen remodeling contributes to, but does not fully account for, the activity of SilMA@L-Leu [72]. This mechanistic framework links material composition, extracellular matrix remodeling, and bone regeneration and may guide the further optimization of silk fibroin-based scaffolds for bone tissue engineering [24].

## 5 Conclusions

In this study, we developed a photocurable, 3D-printable SilMA hydrogel scaffold incorporating L-Leu. L-Leu incorporation preserved printability and enabled the fabrication of structurally defined porous scaffolds with favorable mechanical support, interfacial hydration, and cytocompatibility. In vitro, SilMA@L-Leu promoted osteogenic differentiation of BMSCs and enhanced HUVEC migration and angiogenic activity, demonstrating parallel enhancement of osteogenic and angiogenic responses. These findings do not, however, establish causal coupling between osteogenic and endothelial cells.

In a rat critical-size calvarial defect model, SilMA@L-Leu (300 ng mL^−1^) promoted new bone formation and improved microstructural parameters at both 6 and 12 weeks. Histological analyses further demonstrated enhanced collagen deposition and accelerated mineral maturation, with overall repair performance superior to that of the Control group and, for some parameters, approaching that of the Allograft group. Mechanistically, proteomic analysis identified enrichment of ECM-remodeling-related pathways and highlighted PLOD2 as a candidate collagen-remodeling node. siPLOD2 attenuated collagen-associated ECM remodeling and osteogenic gene activation, suggesting that PLOD2-associated collagen matrix remodeling may contribute to the pro-osteogenic activity of SilMA@L-Leu. Taken together, this cell-free 3D-printed scaffold integrates structural design with matrix-regulatory bioactivity and represents a promising biomaterial strategy for bone defect repair. Future studies should further optimize long-term mechanical stability and release behavior and perform comprehensive safety assessments, including hematological, biochemical, immunological, biodistribution, and longer-term toxicity analyses in higher-order animal models.

## CRediT authorship contribution statement

**QiaoYu Zhang:** Writing – original draft, Supervision, Data curation, Conceptualization. **ZiJie An:** Validation, Software, Resources, Methodology. **Lianzong Hang:** Validation, Methodology, Investigation, Formal analysis. **JingYu Liu:** Visualization, Software, Investigation, Data curation, Conceptualization. **Chen Gu:** Supervision, Methodology, Formal analysis, Data curation. **Jia Zhu:** Validation, Methodology, Formal analysis. **YaWei Zhang:** Visualization, Methodology, Formal analysis. **Lei Wang:** Software, Investigation, Formal analysis. **Wenhui Hu:** Validation, Data curation, Conceptualization. **Tianming Wang:** Supervision, Formal analysis, Conceptualization. **Xin Zhang:** Supervision, Resources. **Yue Mao:** Resources, Methodology, Formal analysis, Conceptualization. **Rui Zhao:** Investigation, Funding acquisition, Formal analysis, Conceptualization. **Yongqiang Zhang:** Validation, Resources, Formal analysis, Data curation. **Kun Zhu:** Writing – review & editing, Writing – original draft, Visualization, Validation, Supervision, Software, Resources, Project administration, Methodology, Investigation, Funding acquisition, Formal analysis, Data curation, Conceptualization.

## Ethics approval

The experimental protocol was established, according to the ethical guidelines of the Helsinki Declaration and was approved by the Ethics Committee of The First Affiliated Hospital of Bengbu Medical College([2024]KY032).

## Financial support and sponsorship

This study was supported by the Clinical and Translational Research Project of Anhui Province (202527c10020116), the Natural Science Research Project of Anhui Educational Committee (2024AH051233, 2025AHGXZK31546), the Science Research Project of Anhui Health Commission (AHWJ2023A30070), the Medical Innovation Foundation from Spinal Deformity Clinical Medicine and Research Center of Anhui Province (AHJZJX-GG2023-004), the Health Research Project of Bengbu City (BBWK2024C205), the Natural Science Research Project of Bengbu Medical University (2024byzd129), The "Challenge and Response" Scientific Research Project of Bengbu Medical University(2025byjbgs036).

## Declaration of competing interest

The authors declare no conflict of interest.

## Data Availability

Data will be made available on request.
